# A novel lncRNA *Discn* fine-tunes replication protein A (RPA) availability to promote genomic stability

**DOI:** 10.1038/s41467-021-25827-6

**Published:** 2021-09-22

**Authors:** Lin Wang, Jingzheng Li, Hu Zhou, Weidao Zhang, Jing Gao, Ping Zheng

**Affiliations:** 1 State Key Laboratory of Genetic Resources and Evolution, Kunming Institute of Zoology, Chinese Academy of Sciences, Kunming, Yunnan 650223 China; 2 Yunnan Key Laboratory of Animal Reproduction, Kunming Institute of Zoology, Chinese Academy of Sciences, Kunming, Yunnan 650223 China; 3 University of Chinese Academy of Sciences, Beijing, 101408 China; 4 Department of Analytical Chemistry and CAS Key Laboratory of Receptor Research, Shanghai Institute of Materia Medica, Chinese Academy of Sciences, Shanghai, 201203 China; 5 KIZ/CUHK Joint Laboratory of Bioresources and Molecular Research in Common Diseases, Kunming Institute of Zoology, Chinese Academy of Sciences, Kunming, Yunnan 650223 China; 6 Center for Excellence in Animal Evolution and Genetics, Chinese Academy of Sciences, Kunming, 650223 China

**Keywords:** DNA replication, Stem cells

## Abstract

RPA is a master regulator of DNA metabolism and RPA availability acts as a rate-limiting factor. While numerous studies focused on the post-translational regulations of RPA for its functions, little is known regarding how RPA availability is controlled. Here we identify a novel lncRNA *Discn* as the guardian of RPA availability in stem cells. *Discn* is induced upon genotoxic stress and binds to neucleolin (NCL) in the nucleolus. This prevents NCL from translocation into nucleoplasm and avoids undesirable NCL-mediated RPA sequestration. Thus, *Discn*-NCL-RPA pathway preserves a sufficient RPA pool for DNA replication stress response and repair. *Discn* loss causes massive genome instability in mouse embryonic stem cells and neural stem/progenigor cells. Mice depleted of *Discn* display newborn death and brain dysfunctions due to DNA damage accumulation and associated inflammatory reactions. Our findings uncover a key regulator of DNA metabolism and provide new clue to understand the chemoresistance in cancer treatment.

## Abstract (web summary)

Various lncRNAs functions have been associated to replication stress and DNA double strand breaks in ESC. Here the authors reveal the lncRNA *Discn*-NCL-RPA axis pathway, which can regulate the availability of RPA pool for DNA replication stress response and repair.

## Introduction

Stem cells (SCs, including pluripotent embryonic stem cells and tissue stem cells) are cellular basis for organism development and tissue homeostasis. Genetic lesions in SCs usually induce the loss of stem cell identity, causing numerical or functional perturbations in SCs. These perturbations can have catastrophic consequences for tissue and organismal homeostasis, resulting in embryonic lethality, developmental defects, degenerative disorders, and oncogenesis^1^. In adaptation to their functional significance, SCs are able to maintain superior stable genome. The genome maintenance ability in SCs lies in their unique DNA metabolic properties in aspects of DNA replication and repair. Limited pioneer works showed that mouse embryonic stem cells (mESCs) utilize ZSCAN4-mediated DNA recombination-based pathway to lengthen the telomere^2^. ESCs do not use TRF2 to protect the telomere, whereas TRF2 is essential for telomere protection in somatic cells^3,4^. Compared to somatic cells, ESCs have superior abilities to resolve replication stress and repair DNA damages. Several stem cell-specific proteins, including SALL4, FILIA, and FILIA-FLOPED protein complex were identified as the underlying regulators^5–7^. Despite these progresses, how SCs efficiently replicate and repair DNA during frequent cell proliferation still remains largely unclear. Comprehensive elucidation of the underpinning mechanisms would largely expand our knowledge on the regulations of DNA metabolism and genomic stability maintenance in stem cells, and help understand the relevant pathophysiological conditions^1^.

During DNA metabolism including DNA replication, repair and recombination, single-stranded DNA (ssDNA) is frequently generated and rapidly coated by replication protein A (RPA) complex. RPA is composed of three subunits RPA70, RPA32, and RPA14, and is the major ssDNA-binding protein in eukaryotic cells. RPA binding to ssDNA not only protects the naked ssDNA from nucleolytic degradation and prevents the secondary struction formation in ssDNA, but also serves as a platform to launch downstream events in all DNA metabolic processes. Thus, RPA acts as a master regulator in DNA metabolism. RPA perturbations, for instance RPA haploinsufficiency, depletion, or exhaustion, can result in DNA replication catastrophe, DNA repair defects and genomic instability. Given its central roles, RPA has emerged as a focus for cellular responses to genotoxic stress^8–10^. To date, the majority of studies investigated the regulations of RPA functions in different aspects of DNA metabolism, and revealed the post-translational modifications including phosphorylation, SUMOylation, and ubiquitination as important regulators^10^. Compared to post-translational modifications, however, little is known on how cells regulate RPA availability, which is a rate-limiting factor in RPA functions.

DNA replication stress and DNA DSBs can generate massive ssDNA, which rapidly exhausts RPA reservoir. Increasing RPA availability is essential to improve cellular resistence to these stresses. Compared to somatic cells, ESCs are more tolerant to DNA DSBs and replication stress^7,11^. This raised a hypothesis that ESCs may develop unique strategies to better sustain free RPA pool. In this study, we aimed to understand the mechanisms regulating the resistence of ESCs to DNA replication stress and DNA DSBs. We focused on long noncoding RNAs (lncRNAs), which can modulate and fine-tune gene expression and protein functions through interacting with DNA, RNAs, and proteins in many cellular events^12^. By comparing the RNA expression profiles of mouse ESCs and their differentiated progenies under unperturbed and genotoxic treatment conditions, we identified a short list of lncRNAs that were specifically induced in ESCs upon both replication stress and DNA DSBs. We then characterized in detail an un-annotated lncRNA *Discn* (DNA damage-induced stem cell specific noncoding RNA), whose expression displayed the most robust increase in response to DNA replication stress and DSBs. Our results showed that *Discn* ensured sufficient RPA availability via *Discn*-NCL-RPA pathway to promote genome stability. Notably, knockout of *Discn* in mice caused newborn death as well as brain dysfunctions, demonstrating its important physiological functions in normal embryonic development.

## Results

### A novel lncRNA *Discn* responds to genotoxic stress in stem cells

**Fig. 1:**
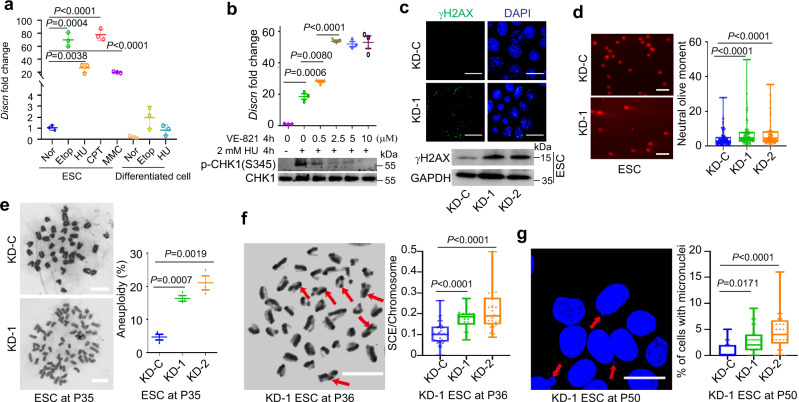
*Discn* safeguards genomic stability of mouse ESCs. **a** Mouse ESCs and their differentiated isogenic cells were cultured in normal condition (Nor) or treated with 2 mM hydroxyurea (HU), 10 μM etoposide (Etop), 1 μM camptothecin (CPT), or 8 μg/mL mitomycin C (MMC) for 4 h. The relative expression level of *Discn* was determined by quantitative RT-PCR. The amount in ESCs under normal condition was set as 1.0. *n* = 3 biologically independent samples. Data were presented as mean ± SEM, two-tailed unpaired *t* test. **b** Quantitative RT-PCR showed *Discn* expression was increased by HU-induced replication stress. Blockage of ATR signaling by inhibitor VE-821 further stimulated the expression of *Discn* in a dose-dependent manner. The *Discn* amount in ESCs under normal condition was set as 1.0. Upper panel showed the *Discn* expression fold changes and lower panel was the validation of ATR inhibition by VE-821. *n* = 3 biologically independent samples, two-tailed unpaired *t* test. Data were presented as mean ± SEM. **c** Compared to control (KD-C), knockdown of *Discn* by two independent shRNAs (KD-1 and KD-2, respectively) increased DNA double-strand breaks (DSBs) as monitored by the increased level of γH2AX. Upper panel showed the representative immunostaining images. Lower panel showed the immunoblotting result. Three independent experiments were repeated with similar results. Scale bar, 20 μm. **d** Neutral comet assay validated the increased DNA DSBs in *Discn* KD cells. Left panel, the representative images. Right panel, the quantification of comet tail length. At least 100 tails were analyzed in each group in three independent replications. Scale bar, 400 μm. **e** *Discn* KD ESCs had higher rate of aneuploidy. Representative image at passage 35 (P35) (Left panel) and the quantification (right panel) were obtained from at least 50 metaphase spreads in three independent replications. Data were shown as mean ± SEM, two-tailed Student’s *t*-test. Scale bar, 10 μm. **f** *Discn* KD increased the sister chromatid exchange rates. Left panel showed the representative images of sister chromatid exchange (arrows) in KD ESCs at passage 36. Quantification was shown on the right. *n* = 3 biologically independent samples, at least 20 metaphase spreads were analyzed in each group. Scale bar, 10 μm. **g** *Discn* KD increased micronuclei formation. Left panel showed the representative images of micronuclei (arrows). Scale bar, 20 μm. Right panel showed the percentages of cells with micronuclei. At least 25 visual field containing 1000 cells were analyzed in three replications. **d**, **f**, **g** Data were representative of individual values with box and whiskers plots showing the median, upper and lower quartiles, and minimum and maximum. Two-tailed Student’s *t*-test.

To explore if there exist unique lncRNAs responsive to DNA replication stress and DNA DSBs in stem cells, we performed RNA-seq and compared the genome-wide RNA expression profiles of mouse ESCs to their differentiated progenies cultured under unperturbed or genotoxic conditions (hydroxyurea or etoposide treatment)^13^. The cellular identity of differentiated progenies was validated by the expression levels of pluripotency genes and three germ layer markers (Supplementary Fig. 1a). We identified a short list of lncRNAs (Supplementary Fig. 1b), which were predominantly expressed in ESCs and significantly stimulated by genotoxic treatments. Among them, an un-annotated lncRNA displayed the most robust response to genotoxic stress, with expression level increasing more than twenty fold after hydroxyurea, etoposide, mitomycin C, or camptothecin treatment (Fig. 1a). We, therefore, named this lncRNA as *Discn* (DNA damage-induced stem cell specific noncoding RNA). We also treated ESCs with hydroxyurea plus ATR inhibitor VE-821, which can robustly enhance the unscheduled origin firing and ssDNA formation under replication stress. Intriguingly, inhibition of ATR signaling during replication stress further stimulated the expression of *Discn* in a dose-dependent manner (Fig. 1b). This expression pattern suggested that *Discn* was responsive to the generation of ssDNA, and might be involved in regulation of genomic stability via ssDNA-related events.

To better understand *Discn*, we obtained its full length sequence (GenBank accession number: MZ269527) using 3ʹ- and 5ʹ -end RACE (rapid amplification of cDNA ends) (Supplementary Fig. 1c). *Discn* is located on chromosome 11 and contains two exons (Supplementary Fig. 1d). Although several potential coding frames are present in the sequence (Supplementary Fig. 1c), they did not translate into proteins or peptides (Supplementary Fig. 1e). Thus, *Discn* is a potential noncoding RNA. We also observed the expression of *Discn* in several available mouse tissue stem/progenitor cell samples (e.g., cultured mouse spermatogonia stem cells, mouse neural stem/progenitor cells (NSPCs), mammary stem cells) (Supplementary Fig. 1f). However, *Discn* was not detected in many organs except of brain and testis (Supplementary Fig. 1g). In addition, *Discn* is conserved among mammalian species (Supplementary Fig. 1h). These data collectively proposed a potential and probably a general role of *Discn* in regulating stem cell genomic stability.

### *Discn* safeguards genomic stability of cultured stem cells

To find out if *Discn* is required to maintain genomic stability of stem cells, we first evaluated its functions in in-vitro cultured stem cells. *Discn* was efficiently knocked down (KD) via two independent short hairpin RNAs (shRNAs) in mESCs (Supplementary Fig. 1i). Under unperturbed culture condition, *Discn* KD mESCs displayed elevated level of DNA DSBs, as monitored by increased amount of γH2AX (Fig. 1c). The severe DNA DSBs were further validated by neutral comet assay (Fig. 1d), which measures the DSBs at single cell resolution^14^. In addition, many chromosome instability (CIN) phenotypes including aneuploidy (Fig. 1e), chromosome translocation measured by sister chromatid exchange (SCE) (Fig. 1f), and micronuclei formation (Fig. 1g), were prevalent in *Discn* KD mESCs. However, the mRNA expressions of core pluripotency regulators *Nanog*, *Klf4*, *Sox2*, and *Oct4* were not affected (Supplementary Fig. 1j). Consistently, the mutant mESCs displayed normal morphology (Supplementary Fig. 1k), although their proliferation rates were reduced (Supplementary Fig. 1l).

We also examined the role of *Discn* in cultured mouse NSPCs. After knocking down *Discn* (Supplementary Fig. 2a), we consistently observed the severe genome instability in NSPCs (Supplementary Fig. 2b–e). Thus, these results collectively demonstrated a general and critical function of *Discn* in ensuring genome stability of in-vitro propagated stem cells.

### *Discn* regulates replication stress response and HR-mediated DNA repair

**Fig. 2:**
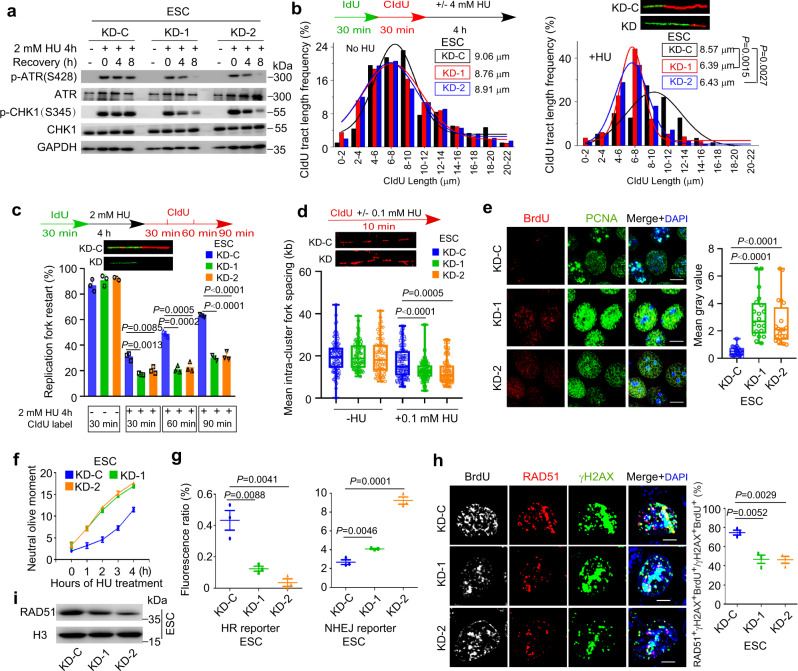
*Discn* regulates DNA replication stress response and HR-mediated DNA DSB repair. **a** Mouse ESCs were treated with hydroxyurea (HU) to induce replication stress. ATR-CHK1 kinase activation failed to be well sustained in two *Discn* knockdown cells (KD-1 and KD-2) when compared to the knockdown control (KD-C). Three independent experiments were repeated with similar results. **b** DNA fiber assay revealed that the stalled forks in *Discn* KD cells were prone to undergo degradation after HU treatment. 200 fibers from three independent replications were analyzed. **c** *Discn* KD compromised the stalled fork restart. At least 200 fibers from three independent replications were analyzed. **d** *Discn* KD enhanced dormant origin firing as indicated by the reduced mean fork spacing. At least 50 continuous fibers from three independent replications were analyzed. **e** *Discn* KD increased the content of ssDNA as measured by the native BrdU incorporation assay at S phase. Scale bar, 10 μm. Quantification is shown on the right. At least 20 images were analyzed in each condition. **f** Compared to control ESCs (KD-C), *Discn* KD cells were more sensitive to hydroxyurea treatment and accumulated more DNA DSBs as measured by neutral comet assay. Data were from three replications and shown as mean ± SEM. **g** FACS analysis of reporter ESCs showed that *Discn* KD suppressed HR-mediated DNA DSB repair, but inversely stimulated NHEJ repair pathway. Data were from three independent experiments. **h** DNA DSBs were generated by laser microirradiation. *Discn* KD impaired the recruitment of Rad51 to DSB sites labeled with γH2AX at S phase (BrdU^+^), indicating the suppression of HR pathway. Right panel showed the proportions of S phase cells capable of HR repair (three replicates, 50 cells in each replicate). Scale bar, 5 μm. **i** Immunoblotting showed a decrease in chromatin-bound Rad51 in *Discn* KD ESCs synchronized at S phase. Three independent experiments were repeated with similar results. **b**, **c**, **f**–**h** Data were shown as mean ± SEM, two-tailed Student’s *t*-test. **d**, **e** Data were representative of individual values with box and whiskers plots showing the median, upper and lower quartiles, and minimum and maximum. Two-tailed Student’s *t*-test.

We next went on to elucidate the functions and mechanisms of *Discn* using mouse ESC as a model. Endogenous DNA DSBs frequently arise when the DNA replication and/or repair are compromised. In response to replication stress, cells elicit coordinated reactions, which locally protect and repair the stalled forks, and globally inhibit cell cycle progression and dormant origin firing in order to avoid excessive ssDNA generation^15^. To find out if *Discn* depletion compromises DNA replication stress response, we induced fork stalling by hydroxyurea treatment and examined the local and global responses of cells. ATR-CHK1 signaling plays a central coordination role in replication stress response. In WT ESCs, ATR kinase was robustly activated and efficiently sustained, as monitored by the persistent phosphorylation of its downstream effector CHK1 at Ser345^16^. However, ATR signaling failed to be well maintained when *Discn* was lost, although its initial activation was grossly normal (Fig. 2a). We then utilized DNA fiber assay to closely examine the behaviors of replication forks^17^. Compared to *Discn* proficient cells, the stalled forks in *Discn* KD cells were prone to degradation under hydroxyurea treatment (Fig. 2b) and difficult to restart after release from stress (Fig. 2c). This indicated that the local responses to protect/repair stalled forks were compromised when *Discn* was absent. Notably, more dormant replication origins were fired (Fig. 2d) and significantly higher level of ssDNA was detected in *Discn* KD ESCs following hydroxyurea treatment (Fig. 2e), demonstrating that the global response to suppress unscheduled origin firing was also impaired by *Discn* KD. As a result, *Discn* KD ESCs were less tolerant to replication stress and produced more DSBs at different time-points of hydroxyurea treatment when compared to *Discn* proficient ESCs (Fig. 2f).

Because *Discn* expression was also induced by etoposide treatment which generates DNA DSBs^13^, we examined its possible involvement in DSB repair. DNA DSBs are repaired by two major pathways: homologous recombination (HR) and classical nonhomologous end-joining (NHEJ) pathways, both of which can be monitored through engineered GFP-based reporters^18^. In the reporter cells, site-specific DSBs are generated in a cassette after expression of endonuclease I-SceI. Repair of the DSBs by respective HR or NHEJ pathway restores the functional RFP or GFP gene and the numbers of RFP- or GFP-expressing cells counted by flow cytometry provide quantitative measure of HR or NHEJ efficiency. To this end, we efficiently knocked down *Discn* in these reporter mESC lines by using the aforementioned shRNAs, and measured the respective HR and NHEJ repair efficiency. In *Discn* KD cells, HR repair was suppressed, whereas NHEJ pathway was inversely stimulated probably due to repair compensation (Fig. 2g). To further validate the impairment of HR pathway, we examined the recruitment of recombinase RAD51 to DSB sites, which is essential for HR repair^19^. Following laser micro-irradiation, much less *Discn* KD cells recruited RAD51 to DSB sites (labeled with γH2AX) at S-phase of cell cycle when compared to WT ESCs (Fig. 2h). In concordance, the amounts of chromatin-bound RAD51 proteins were significantly reduced by *Discn* KD in ESCs synchronized at S phase and treated with etoposide (Fig. 2i). Taken together, these data support that *Discn* regulates DNA replication stress response and HR-mediated DNA DSB repair.

### *Discn* accumulates in nucleolus and binds to nucleolin (NCL)

**Fig. 3:**
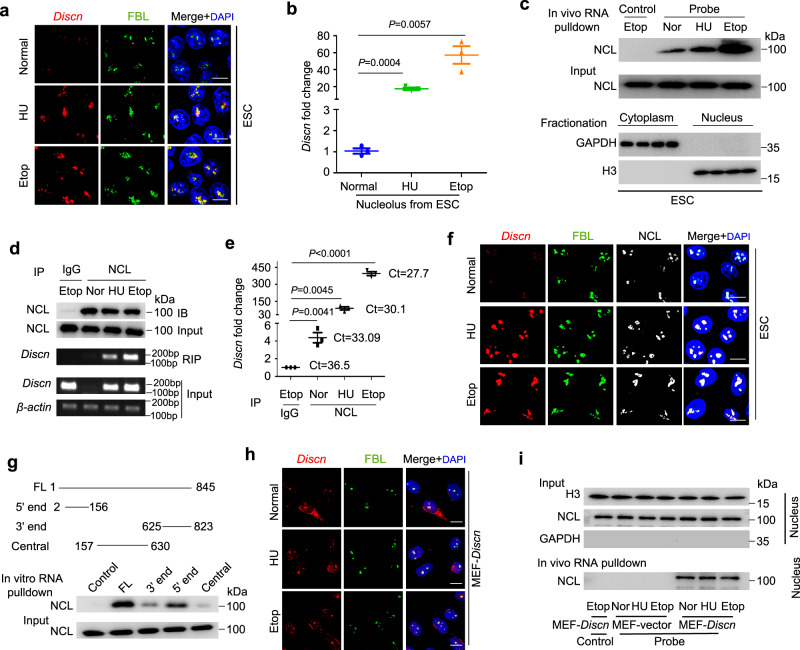
*Discn* localizes in nucleolus and interacts with nucleolin (NCL). **a** mESCs were cultured under normal condition or treated with 2 mM hydroxyurea (HU) or 10 μM etoposide (Etop) for 4 h. RNA-FISH detected the localization of *Discn* in nucleolus labeled with fibrillarin (FBL) upon treatments. Three independent experiments were repeated with similar results. **b** Nucleolus was fractionated and quantitative RT-PCR showed the increased accumulation of *Discn* in nucleolus after HU or etoposide treatment. rRNA was used as control to calculate the relative expression of *Discn*. The amount of *Discn* in nucleolus of ESCs under normal condition was set as 1.0. Data were shown as mean ± SEM from three replications. Two-tailed Student’s *t*-test. **c** ESCs were fractionated into compartments of cytoplasm and nucleus (lower panel). In vivo RNA pulldown combined with immunoblotting on nuclear fraction detected the *Discn*-NCL association under normal or genotoxic treatment conditions (upper panel). Note that treatments did not affect NCL expression (middle panel). Pulldown assay using sense probe was set as negative control. Three independent experiments were repeated with similar results. **d** RNA immunoprecipitation with NCL antibody confirmed the *Discn*-NCL association. Upper panel showed NCL itself was successfully precipitated. Middle panel showed the RT-PCR detection of *Discn* precipitated with NCL. Lower panel showed the input. Three independent experiments were repeated with similar results. **e** The relative levels of *Discn* immunoprecipitated with NCL were determined by quantitative RT-PCR. *n* = 3 biologically independent samples, two-tailed unpaired t test. Data were presented as mean ± SEM. **f** Co-localization of *Discn* and NCL in nucleolus labeled with FBL in mESCs under normal or treatment conditions. Three independent experiments were repeated with similar results. **g** In vitro RNA pulldown assay revealed that *Discn* fragment containing 5ʹ end displayed high affinity to NCL. FL, full length *Discn*. Control, antisense *Discn*. Three independent experiments were repeated with similar results. **h** Co-immunostaining showed the localization of *Discn* in nucleolus of MEFs ectopically expressing *Discn* (MEF-*Discn*). Note that HU or etoposide treatment did not further enhance the accumulation of *Discn* in nucleolus. Three independent experiments were repeated with similar results. **i** MEFs with ectopic expression of *Discn* or transfected with vector (MEF-vector) were fractionated into cytoplasmic and nuclear compartments (upper panel). In vivo RNA pulldown using nuclear fraction validated the *Discn*-NCL association (lower panel). Note that compared to normal condition (Nor), HU or etoposide treatment did not further stimulate the *Discn*-NCL interaction. Sense probe RNA pulldown was set as negative control. Three independent experiments were repeated with similar results. Scale bar, 10 μm.

To understand how *Discn* regulates replication stress response and HR-mediated DNA repair, we first investigated its subcellular localization under normal and stressed conditions by RNA fluorescence in situ hybridization (RNA-FISH). No specific distribution pattern of *Discn* was detected when using conventional staining procedures. However, following extraction of soluble *Discn* with Triton X-100, we observed faint staining of *Discn* in nucleolus labeled with fibrillarin (FBL) under normal culture condition. Hydroxyurea treatment significantly stimulated the accumulation of *Discn* in nucleolus (Fig. 3a). Fractionation of nucleolus (Supplementary Fig. 3a) followed by quantitative RT-PCR examination verified the increased amount of *Discn* in nucleolus after hydroxyurea treatment (Fig. 3b). Of note, *Discn* exhibited the same subcellular localization in response to etoposide treatment (Fig. 3a, b), suggesting that a common mechanism underlies the dual regulations of *Discn* on replication stress response and HR-mediated DNA repair.

Next, we performed in vitro RNA pulldown followed by mass spectrometry analysis to identify potential interaction proteins of *Discn* (Supplementary Fig. 3b). Among the list of candidates, a nucleolus protein nucleolin (NCL) showed relatively high score (Supplementary Fig. 3c). Immunoblotting examination of NCL on in vitro RNA pulldown samples validated the association of *Discn* with NCL (Supplementary Fig. 3d). Because in vitro binding can yield false-positive interaction, we performed in vivo cross-linking followed by RNA pulldown to validate their interaction. Notably, while weak interaction was detected in nuclear fraction under unperturbed condition, strong association was observed after hydroxyurea or etoposide treatment (Fig. 3c). To further confirm the association of *Discn* with NCL, we conducted RNA immunoprecipitation to examine whether *Discn* can be co-immunoprecipitated with NCL under native condition. As shown, very few *Discn* RNAs were detected in immunoprecipitated samples in unperturbed condition. However, genotoxic treatments robustly increased the amount of *Discn* associated with NCL (Fig. 3d, e). In line with their association, immunostaining showed the co-localization of *Discn* with NCL in nucleolus upon genotoxic stress (Fig. 3f). Collectively, these data support that *Discn* binds to NCL in nucleolus and their association is enhanced by genotoxic stress.

We went on to map the NCL binding sites on *Discn*. NCL has binding motif in target RNAs. The motif is G-rich and has stem-loop secondary structures. NCL is able to associate with RNAs bearing this motif in the absence of other proteins^20^. Using RegRNA 2.0 online tools (http://regrna2.mbc.nctu.edu.tw/index.html)^21^, we identified several putative NCL binding sites predominantly at the 5ʹ end of *Discn*. We then constructed the *Discn* mutants containing either the 5ʹ end (2–156 bp), 3ʹ end (625–823 bp), or central part (157-630 bp). In vitro RNA pull down assay revealed that the NCL binding sites were predominantly localized at the 5ʹ end (Fig. 3g).

### *Discn*-NCL interaction does not require genotoxic stimuli

Genotoxic stress stimulated the expression of *Discn* as well as its association with NCL. To elucidate whether the enhanced *Discn*-NCL interaction was simply due to the increased *Discn* expression or required genotoxic stimuli, we stably expressed *Discn* in WT mESCs (Supplementary Fig. 3e) and re-examined the *Discn*-NCL association. Intriguingly, under unperturbed culture conditions *Discn* localized in nucleolus (Supplementary Fig. 3f) and associated with NCL as detected by in vivo RNA pulldown (Supplementary Fig. 3g). Notably, genotoxic treatment did not further increase their association (Supplementary Fig. 3g).

To gain more evidence, we ectopically expressed *Discn* in mouse embryonic fibroblasts (MEFs) (Supplementary Fig. 3h). Concordantly, *Discn* appeared in nucleolus of MEFs (Fig. 3h) and associated with NCL as detected by in vivo RNA pulldown assay (Fig. 3i). Of note, the association was not affected by genotoxic treatments (Fig. 3i). Thus, localization of *Discn* in nucleolus as well as *Discn*-NCL interaction does not require the genotoxic signaling and rather it seems cell autonomous.

### *Discn* sequesters NCL in nucleolus to preserve the free RPA pool

NCL predominantly resides in nucleolus under unperturbed conditions. However, two studies on somatic cells reported that upon heat shock or genotoxic stress, part of the nucleolar NCL pool translocates into the nucleoplasm, where it binds to RPA^22,23^. The formation of NCL-RPA protein complex in nucleoplasm reduces the free RPA level, thereby counteracting the RPA functions.

**Fig. 4:**
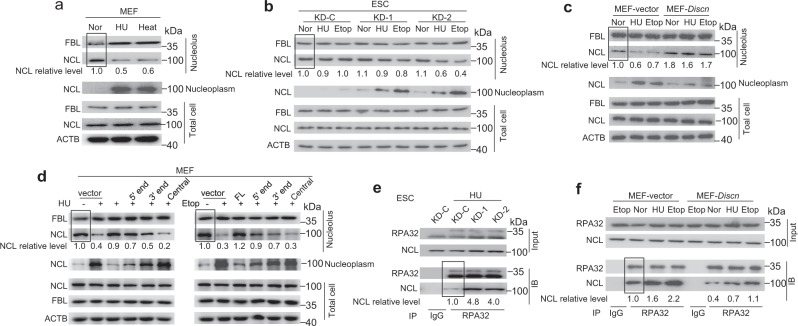
*Discn* sequesters NCL in nucleolus to prevent NCL-RPA association in nucleoplasm. **a** Nucleolus and nucleoplasm were fractionated in MEFs. Immunoblotting revealed that HU or heat treatment caused robust translocation of NCL from nucleolus to nucleoplasm. **b** Fractionation combined with immunoblotting showed that nucleoplasmic NCL level remained comparable before and after genotoxic treatment in *Discn* proficient (KD-C) mESCs. However, in *Discn* knockdown ESCs (KD-1, KD-2) genotoxic treatment evoked a robust increase of NCL level in nucleoplasm accompanied with a mild NCL decrease in nucleolus. **c** Ectopic expression of *Discn* in MEFs attenuated the translocation of NCL from nucleolus to nucleoplasm. **d** Different *Discn* fragments (5ʹ end, 3ʹ end, and central part) expressed in MEFs showed distinct efficiency to prevent the translocation of NCL from nucleolus to nucleoplasm. **e** Co-immunoprecipitation (Co-IP) showed that *Discn* KD in mESCs increased the association of NCL with RPA32. **f** Similarly, ectopic expression of *Discn* in MEFs suppressed the interaction of NCL with RPA32. Cells were cultured under normal condition (Nor) or treated with HU (2 mM, 4 h), etoposide (10 μM, 4 h), or heat shock (44 °C, 4 h). In (**a**–**d**), NCL relative levels in nucleolus were normalized by FBL. In (**e**, **f**), NCL relative levels were normalized by co-immunoprecipitated RPA32. The level in samples marked with box was set as 1. All experiments were repeated three times with similar results.

Based on the above knowledge, we wondered whether *Discn* regulates genomic stability by targeting the NCL-RPA complex. To this end, we first evaluated the functional significance of *Discn*-NCL interaction in nucleolus. In line with previous reports, hydroxyurea or heat stress did not change NCL protein expression in MEFs. However, these treatments drastically increased the relocation NCL into nucleoplasm, and conversely decreased the retention of NCL in nucleolus, as determined by the fractionation of nucleolus and nucleoplasm combined with immunoblotting analysis (Fig. 4a). Intriguingly, we observed a distinctive pattern of NCL distribution in mESCs, in which the NCL protein level in nucleoplasm remained comparable before and after genotoxic treatments (Fig. 4b). Concordantly, NCL retention in nucleolus was not obviously affected by genotoxic stress (Fig. 4b). Thus, unlike in differentiated cells, genotoxic stress does not evoke obvious NCL translocation from nucleolus to nucleoplasm in mESCs.

We then examined if *Discn* loss influenced NCL distribution in mESCs. Unlike in *Discn* proficient ESCs, genotoxic treatment caused part of the nucleolar NCL pool relocation to nucleoplasm in *Discn* KD ESCs, as manifested by a substantial increase of NCL level in nucleoplasm. Due to the high abundance of NCL in nucleolus, release of part of the nucleolar NCL pool into nucleoplasm caused a mild but visible reduction of NCL level in nucleolus (Fig. 4b). The translocation of NCL from nucleolus to nucleoplasm in *Discn* KD ESCs was further verified by immunofluorescence analysis (Supplementary Fig. 3i). Concordantly, ectopic expression of *Discn* in MEFs significantly attenuated the translocation of NCL from nucleolus to nucleoplasm (Fig. 4c). We also expressed the three *Discn* fragments (5ʹ end, 3ʹ end, and central part), respectively, in MEFs and examined their influence on NCL translocation. In line with their ability to bind to NCL (Fig. 3g), 5ʹ end fragment displayed the higher efficiency to prevent NCL translocation to nucleoplasm than 3ʹ end, whereas central part had no effect (Fig. 4d). These data altogether supported that *Discn*-NCL interaction sequestered NCL in nucleolus.

In somatic cells, NCL forms protein complex with RPA in nucleoplasm^23^. Given that the NCL protein level increased drastically in nucleoplasm of *Discn* KD ESCs (Fig. 4b), we speculated that NCL-RPA association was subsequently enhanced. Indeed, co-immunoprecipitation using antibody against RPA32, one of the RPA subunits^24^, revealed a significant induction of NCL-RPA32 interaction in *Discn* KD mESCs compared to *Discn* proficient ESCs upon hydroxyurea treatment (Fig. 4e). Conversely, ectopic expression of *Discn* in MEFs significantly decreased the NCL-RPA32 complex formation (Fig. 4f). Because the total RPA protein expression was not influenced by *Discn* depletion (Supplementary Fig. 3j), we concluded that *Discn* KD led to the reduction of free RPA level in ESCs.

### Overexpression of RPA rescues the genomic instability of *Discn* KD cells

**Fig. 5:**
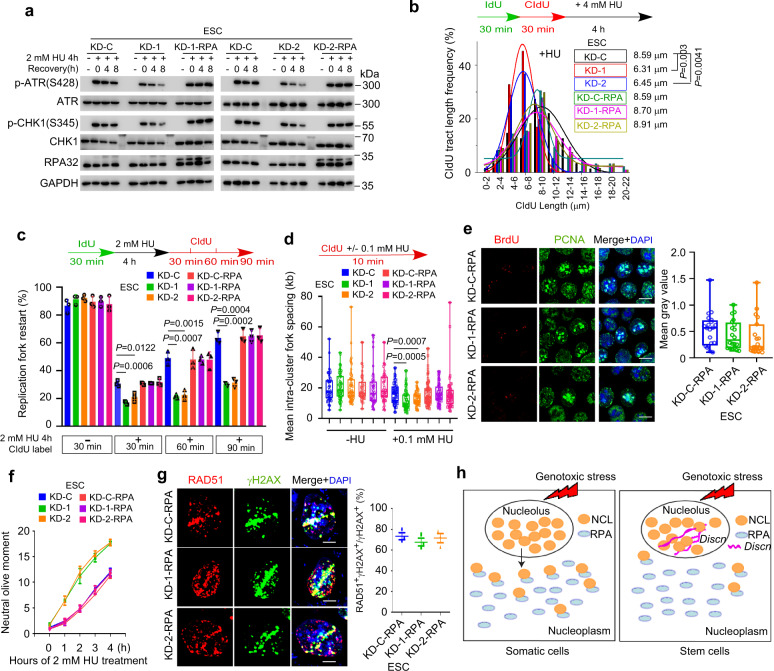
RPA overexpression rescues genomic instability in *Discn* KD mESCs. RPA overexpression in *Discn* KD mESCs restored the ATR-CHK1 signaling (**a**), the stalled fork stability (**b**), and the fork restart ability (**c**). It also rescued the defects in dormant origin firing (**d**), ssDNA generation (**e**), and DSB formations (**f**) under HU treatment. In addition, RPA overexpression in *Discn* KD mESCs recovered the HR repair competence (**g**). Working model of *Discn* in safeguarding free RPA pool and genomic stability (**h**). Scale bar, 10 μm in (**e**) and 5 μm in (**g**). Three independent experiments were repeated with similar results in (**a**). At least 200 fibers from three independent replications were analyzed in (**b**, **c**). At least 50 continuous fibers from three independent replications were analyzed in (**d**). At least 19 images were analyzed in each condition in (**e**). Data were from three replications in (**f**, **g**). Data were representative of individual values with box and whiskers plots showing the median, upper and lower quartiles, and minimum and maximum in (**d**, **e**). Data were presented as mean ± SEM in (**c**, **f** and **g**). Statistical differences were determined using two-tailed Student’s *t*-test.

Because RPA is a master regulator of DNA metabolism and critical guardian of genomic integrity^25,26^, we reasoned that insufficient RPA surplus in *Discn* KD SCs might underpin the genomic instability defects. To test this hypothesis, we over-expressed the three subunits of RPA complex (RPA70, RPA32, and RPA14, respectively) in *Discn* KD ESCs (Supplementary Fig. 4a), and examined if this could rescue the defects. Notably, the cellular responses to hydroxyurea or etoposide treatment were fully restored. These included the ATR-CHK1 signaling (Fig. 5a), the stalled fork stabilization (Fig. 5b) and restart (Fig. 5c), the suppression of unscheduled origin firing (Fig. 5d) and ssDNA formation (Fig. 5e), the tolerance to hydroxyurea-induced replication fork breakage (Fig. 5f), and the HR-mediated DSB repair (Fig. 5g). Concordantly, the overall DNA DSB contents (Supplementary Fig. 4b) and the cell proliferation rates (Supplementary Fig. 4c) of *Discn* KD ESCs were recovered to normal by overexpression of RPAs. We also examined if the same machanism operated in NSPCs. Similar to the observations in mESCs, overexpression of the RPA complex in cultured *Discn* KD NSPCs rescued the genomic instability phenotypes (Supplementary Fig. 5). Collectively, these data support that *Discn*-NCL-RPA pathway functions generally in SCs. Protein expression level also serves as a regulatory layer. We found that ESCs express more RPA proteins than NSPCs and MEFs, whereas NSPCs and MEFs had comparable RPA expression (Supplementary Fig. 4d). Thus, *Discn* adds a fine-tune regulatory layer to RPA surplus.

In conclusion, our data proposed a unique mechanism in SCs, in which lncRNA *Discn* is robustly induced by genotoxic stress and localizes in nucleolus where it interacts with NCL and sequesters NCL in nucleolus. Interaction of *Discn*-NCL in nucleolus reduces the undesirable NCL-RPA association in nucleoplasm, thereby achieving a sufficient RPA surplus crucial for DNA replication stress response and repair (Fig. 5h). In line with this model, ectopic expression of *Discn* in MEFs reduced the NCL-RPA association (Fig. 4f), and conferred protection against genetoxic stress (Supplementary Fig. 4e–i). However, expression of mutant *Discn* fragments which do not efficiently bind to NCL (3ʹ end and central part) failed to protect MEFs against genotoxic damage (Supplementary Fig. 4i). A recent study reported that lncRNAs, which are generally outnumbered by their associated RNA binding proteins (RBPs), can efficiently regulate the activity of interacting RBPs by driving their phase separation^27^. *Discn* was expressed at about 65–160 copies per mouse ESC under genotoxic conditions (Supplementary Fig. 4j). Future works are required to clarify whether lncRNA-driven phase separation underlies *Discn*-mediated regulation.

### *Discn* knockout mice show newborn death and brain dysfunctions

Stem cells are the cellular basis for embryo development and tissue homeostasis. Genomic instability in pluripotent stem cells or tissue stem cells could impair embryo development, cause premature ageing or cancer susceptibility. The phenotypes that we observed in cultured stem cells prompted us to further examine the physiological functions of *Discn*. To this end, we generated *Discn* knockout (KO) mice via CRISPR/Cas-mediated gene targeting strategy (Supplementary Fig. 6a). Two mouse lines from the same targeting design were established and the success of *Discn* KO was validated by Sanger sequencing of PCR-amplified targeting regions (Supplementary Fig. 6b), as well as the loss of *Discn* expression in brain tissues of newborn KO mice (Supplementary Fig. 6c).

**Fig. 6:**
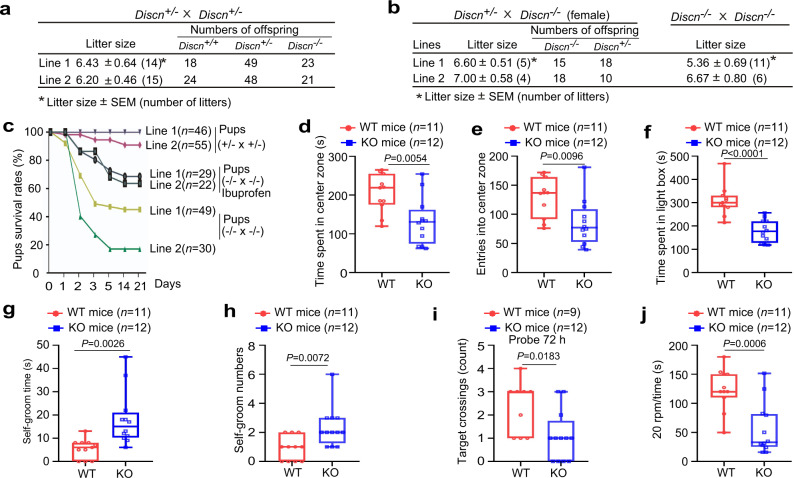
*Discn* knockout (KO) causes newborn death and compromises brain functions in survived adults. **a***Discn*^+/−^ crossing generated pups with normal Mendelian ratios. **b** *Discn*^−/−^ females mated with *Discn*^+/−^ or *Discn*^−/−^ males had normal litter sizes and Mendelian ratios. **c** Survival curve of offsprings from different mating strategies. Note that *Discn*^−/−^ pups from *Discn*^−/−^ crossings displayed newborn death, and administration of ibuprofen (0.4 mg/mL) to pregnant females alleviated the neonate death. Adult *Discn*^−/−^ mice and their WT littermates from *Discn*^+/−^ crossings were utilized for behavioral tests. In open field test, *Discn*^−/−^ mice spent less time in the center zone (**d**) and entered fewer times into the center zone (**e**) compared to the WT littermates. In light–dark box test, *Discn*^−/−^ mice stayed in the light box for less time than WT littermates (**f**). In self-grooming behavior analysis, *Discn*^−/−^ mice spent longer time (**g**) and showed more bouts (**h**) on self-grooming when compared to WT littermates. In Morris water maze test, at 72 h after 7-day probe trials, *Discn*^−/−^ mice showed fewer platform crossings than WT littermates (**i**). In rotarod test, *Discn*^−/−^ mice stayed on the rotating rod for shorter time than WT littermates (**j**). Data were representative of individual values with box and whiskers plots showing the median, upper and lower quartiles, and minimum and maximum in (**d**–**j**). Statistical differences were determined using two-tailed Student’s *t*-test.

Heterozygous mutant mice (*Discn*^+/−^) were viable and no obvious abnormality was observed. Crossings of *Discn*^+/−^ mice in line 1 produced pups with expected Mendelian ratios (Fig. 6a). Similar results were obtained from mouse line 2 (Fig. 6a). To exclude a possible maternal effect of *Discn* on early embryonic development, we mated *Discn*^−/−^ females with *Discn*^+/−^ males. Pups were born in normal Mendelian ratios (Fig. 6b), confirming that loss of *Discn* did not cause embryonic lethality. Consistently, crossing of *Discn*^−/−^ mice produced litters with grassly normal sizes (Fig. 6b). Thus, maternal-zygotic loss of *Discn* does not cause embryonic lethality. However, high rates of newborn death occurred in pups obtained from *Discn*^−/−^ crossings. More than half of them died within one week after birth (Fig. 6c).

We further investigated if *Discn* loss displayed long-term influences on the survived adults. Given that *Discn* was expressed in brain tissue and regulated the genomic integrity of cultured NSPCs, we thus focused on the brain functions. No structural defect was detected in brains of the *Discn*^−/−^ mice no matter whether they were from *Discn*^−/−^ or *Discn*^−/−^ crossings (Supplementary Fig. 6d). We then performed a series of behavioral tests to examine the emotions, learning, spatial memory, and sport performances. Adult littermates (two months old) from *Discn*^+/−^ crossings were utilized in these studies and significant differences were observed between wild-type (WT) and *Discn*^−/−^ mice. *Discn*^−/−^ mice displayed mood and anxiety-related disorders as determined by the following tests. In open field experiment, *Discn*^−/−^ mice spent significantly less time in the center (Fig. 6d) and crossed into the center for fewer times (Fig. 6e) than the WT littermates. Consistently, mutant mice stayed in the light box for shorter time in light–dark box test (Fig. 6f), and had more self-grooming behaviors (Fig. 6g, h) than WT littermates. We next conducted Morris water maze to evaluate the brain functions on learning and spatial memory. *Discn*^−/−^ mice and WT littermates swam in similar speeds (Supplementary Fig. 6e), and had equal ability to find the platform during the seven days of training (Supplementary Fig. 6f, g). However, in the following probe trials in which the escape platform was removed after training and the mice were allowed to search for it in a fixed time period, *Discn*^−/−^ mice displayed fewer platform crossings than WT littermates at 72 h rather than 4 h post training (Fig. 6i, Supplementary Fig. 6h). This result suggested the compromised long-term memory in *Discn* KO mice. We also performed rotarod test to examine the coordination and balance abilities. *Discn*^−/−^ mice stayed on the rotating rod without falling for shorter time than WT littermates when the speed reached 20 rpm (Fig. 6j), indicating the reduced coordination and balance. Because *Discn*^−/−^ neonates generated from *Discn*^−/−^ crossings displayed more severe phenotype than those generated from *Discn*^+/−^ crossings, we suspected that *Discn*^−/−^ adults from *Discn*^−/−^ crossings could have worse brain functions. To test this hypothesis, we compared their coordination and balance abilities. Indeed, *Discn*^−/−^ adults from *Discn*^−/−^ crossings had worse performance than those from *Discn*^+/−^ crossings in rotarod test (Supplementary Fig. 6i). Collectively, our data show that *Discn* plays important physiological functions and its loss in mice causes newborn death as well as brain dysfunctions in survived adults.

### *Discn*^−/−^ mouse brains have massive DNA DSBs and inflammation

**Fig. 7:**
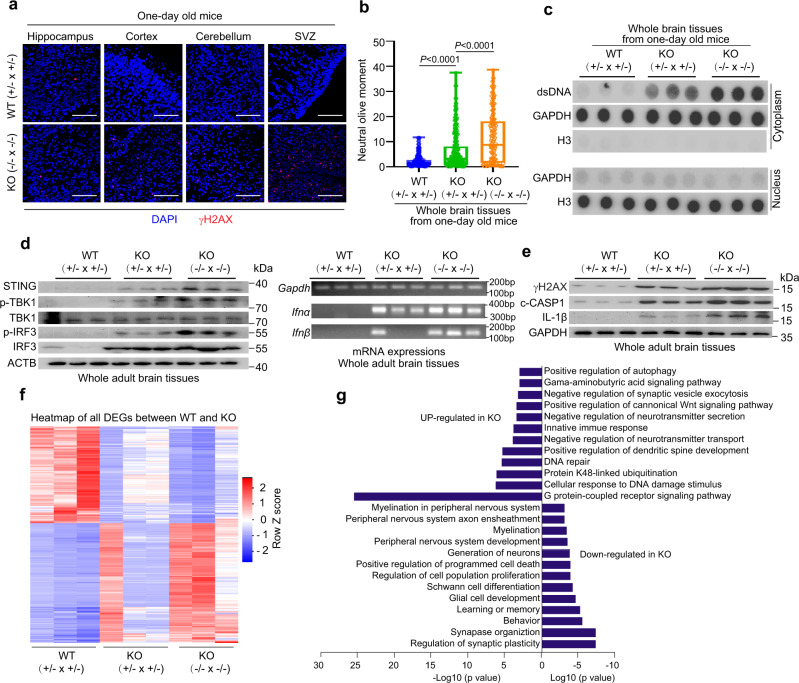
*Discn* knockout (KO) in mice induces massive DNA DSBs and immune reaction. *Discn* KO mouse brains contained higher level of DNA DSBs than WT counterparts, as indicated by γH2AX staining (**a**) and comet assay (**b**). SVZ, subventricular zone. KO (+/− × +/−) indicated the KO mice were from *Discn*^+/−^ crossing, whereas KO (−/− × −/−) indicated the KO mice were from *Discn*^−/−^ crossing. Scale bar, 100 μm. At least 100 tails were analyzed in each comet assay group in three independent replications. Data were representative of individual values with box and whiskers plots showing the median, upper and lower quartiles, and minimum and maximum. Two-tailed Student’s *t*-test. **c** *Discn* KO mouse brains contained higher level of cytosolic dsDNA than WT mice. Mice were 1 day old in (**a**–**c**). **d** The STING pathway was activated in *Discn* KO mouse brains, as indicated by the phosphorylation of TBK1 and IRF3, as well as the induction of type 1 interferon expression. Three independent experiments were repeated with similar results. **e** The inflammasomes were activated in *Discn* KO mouse brains, as indicated by the detection of cleaved caspase 1 (c-CASP1) and mature IL-1β. Three independent experiments were repeated with similar results. **f** Heatmap of differential expressed genes (DEGs) between *Discn* KO and WT counterpart mice brains. **g** Gene ontology (GO) enrichments of DEGs shown in (**f**). Adult mice were used in (**d**–**g**).

NSPCs are the cellular basis for neurogenesis at fetal and postnatal stages. Accumulations of DNA DSBs and cytosolic double-strand DNA (dsDNA) in NSPCs and their progenies not only impaired neurogenesis and brain functions^28,29^, but also evoked innate immune and inflammation responses leading to animal death^30^. Based on the function of *Discn* in in-vitro cultured NSPCs, we hypothesized that *Discn* KO might cause accumulations of DSBs and cytosolic dsDNA in brain cells of neonates and adults, and the damages and associated immune responses underlie the newborn death and brain dysfunctions. Indeed, the brain cells of *Discn*^−/−^ pups (one day old) contained higher level of DNA DSBs measured by γH2AX staining and neutral comet assay (Fig. 7a, b), and cytosolic dsDNA (Fig. 7c) compared to WT pups. Notably, KO pups from *Discn*^−/−^ crossings had more abundant DSBs and cytosolic dsDNA than those from *Discn*^+/−^ crossings (Fig. 7b, c). Similar results were obtained from adult mouse brains (Supplementary Fig. 7a–c).

DNA DSBs and cytosolic dsDNA can activate the STING pathway inducing the type 1 interferon expression and downstream immune responses^31–33^. Cytosolic dsDNA also stimulates the AIM2 inflammasome activation leading to caspase 1 (CASP1) cleavage, pro-interleukin-1β (IL-1β) maturation and cell death via gasdermin D-mediated pyroptosis^31,34^. STING pathway activation is monitored by phosphorylation of the key components TBK1 and IRF3, and the expression of type 1 interferons (INFα and INFβ). Compared to the WT counterparts, adult *Discn* KO mice, in particular those from *Discn*^−/−^ crossings, showed drastic STING activation in brain cells (Fig. 7d). Moreover, cleavage of CASP1 and maturation of IL-1β were elevated in KO mouse brains (Fig. 7e). Intriguingly, the DNA DSBs and immune reactions were not detected in lung tissue of KO mice (Supplementary Fig. 7d). This suggested that the influences of *Discn* loss might be confined to certain tissues/organs where tissue stem cells rely on *Discn* for genome stability. Severe immune and inflammation reactions usually cause animal death. We then asked if the genomic instability-driven inflammation accounted for the newborn death of *Discn* KO mice. To this end, we administrated ibuprofen, which is able to alleviate inflammation, in drinking water to pregnant *Discn*^−/−^ females mated with *Discn*^−/−^ males^35^. Notably, ibuprofen administration partially rescued the defect of newborn death (Fig. 6c), supporting that inflammation is a causative factor for animal death.

We finally examined the whole-genome gene expression changes in *Discn* KO mouse brains by RNA sequencing. Brain tissues of WT and *Discn*^−/−^ mice, which were derived from crossings of *Discn*^+/−^ and *Discn*^−/−^, respectively, were analyzed. We identified 3535 differentially expressed genes (DEGs) whose expressions were consistently decreased or increased (fold change ≥2) in two types of *Discn* KO mice compared to the WT control (Fig. 7f, Supplementary data). Of note, *Discn*^−/−^ mice born from *Discn*^−/−^ crossings displayed the most drastic changes when compared to the other groups. Gene Ontology (GO) term analyses revealed that DEGs down-regulated in *Discn*^−/−^ were mostly involved in the regulations of neurogenesis, neuronal activities and functions (Fig. 7g, Supplementary Fig. 7e, f). DEGs upregulated in *Discn*^−/−^ mice were enriched in terms of negative regulations of neurogenesis and neuronal functions, DNA damage responses, positive regulation of autophagy and cell differentiation (Fig. 7g, Supplementary Fig. 7g). Consistently, term of innate immune response was enriched (Fig. 7g, Supplementary Fig. 7h, i).

## Discussion

Stem cells are the cellular basis for embryo development and tissue homeostasis, and are known to have much more stable genome than differentiated somatic cells. Only recently was it recognized that stem cells employed unique DNA metabolic pathways to efficiently maintain stable genome. Although several pioneer works reported stem-cell specific regulations on telomere lengthening, DNA damage response, and repair^2–7,36^, it remains largely unknown regarding the specificity of DNA metabolism and their relevance to genomic stability in stem cells. LncRNAs play increasingly important functions in many cellular events. Whether lncRNAs are involved in stem-cell specific regulations of DNA metabolism is unexplored. In this study, we identified a list of ESC-specific lncRNAs whose expressions are responsive to genotoxic stress. By analyzing one un-annotated lncRNA *Discn*, we identified a novel regulatory pathway *Discn*-NCL-RPA which preserves a sufficient RPA pool for DNA replication stress response and repair.

During continuous cell proliferation, DNA replication frequently encounters endogenous and exogenous barriers which stall the replication forks and generate replication stress. During stress, ssDNA is persistently generated due to the continuous firing of new origins even the checkpoint is active. The ssDNA is not converted into DNA breaks as long as it is coated and protected by RPA. Thus, the RPA surplus plays a central part and is considered as a common denominator for avoiding replication catastrophe^25,26^. Cells usually express more RPA than needed during the normal unperturbed replication. However, excessive ssDNA generated by replication stress can rapidly exhaust RPA reservoir^26^. Therefore, efficient sustaining the RPA pool is vital to survive the replication stress. Increasing the expression of RPA is one simple strategy that is employed by pluripotent stem cells. Here we reported a novel mechanism in which lncRNA *Discn* sustains the free RPA pool by sequestering NCL in nucleolus and counteracting its association with RPA. The *Discn*-NCL binding in nucleolus seems cell autonomous and does not require stress stimuli. *Discn* is induced by genotoxic stress and its expression level is adjusted by the strength of stress (for instance hydroxyurea treatment plus ATR deficiency). Therefore, *Discn* fine-tunes the RPA reservoir in response to replication stress.

*Discn* depletion causes severe DNA DSBs in cultured mouse ESCs which are in vitro counterparts of the undifferentiated epiblast cells in peri-implantation embryos. Previous study reported that induction of DSBs at gastrulation stage (E6.5-E7.5) could result in apoptosis and embryonic lethality^37^. However, *Discn* KO did not cause prenatal death immediately after gastrulation. This could be due to the fact that the pluripotent epiblast cells exist transiently in vivo (from embryonic E4.5-E5.5) and undergo limited rounds of DNA replication during this time-window. The extent of DNA replication-associated DSBs could therefore be mild and fail to evoke lethal phentotype. In contrast, NSPC-based neurogenesis starts from ~E8.5 and persists throughout the fetal stage and even until after birth. The long time peroids of NSPC proliferation renders the neural system susceptible to the replication-associated DNA damage. Indeed, we detected increased levels of DNA DSB and cytoplasmic dsDNA in brain cells of *Discn* KO mice. Moreover, the DSB level was significantly higher in KO mice derived from *Discn*^−/−^ crossings than in those derived from *Discn*^+/−^ crossings. Concordantly, *Discn* KO mice displayed variable extents of developmental defects ranging from brain dysfunctions to newborn death, depending on the genotypes of their parents.

The genomic instability acted as a causative factor in the developmental defects of *Discn* KO mice. Previous studies including ours have shown that in NSPCs, replication stress-induced DNA DSBs frequently arise in replication fragile sites and form recurrent DSB clusters (RDCs). Many genes critical for neurogenesis in NSPCs harbor RDCs and are susceptible to replication stress-associated perturbations. Thus, replication-associated DNA DSBs in NSPCs directly impacted neurogenesis^29,38,39^. In addition, DNA DSBs and in particular cytoplasmic dsDNA are able to evoke innate immune response and inflammatory reactions^33,40^. Immune reaction alone in developing brain imparts lifelong neuropathology and altered behaviors^41^. Adult neural stem cells are also altered by systemic inflammation^42^. Severe inflammation is able to cause pyroptotic cell death^31,34^. Indeed, *Discn* KO induced the STING pathway activation leading to the production of type-1 interferons. Inflammasomes were also activated in KO brains as evidenced by the cleavage of caspase 1 and maturation of pro-inflammatory cytokine IL-1β. Similar to the tendency of DNA DSB and cytoplasmic dsDNA accumulation, the immune and inflammatory reactions in KO mice from *Discn*^−/−^ crossings were more severe, leading to the newborn death. It is intriguing to know why KO mice from *Discn*^−/−^ crossings accumulated more DNA DSBs than those from *Discn*^+/−^ crossings. One possible explanation is that oocytes and sperms from *Discn*^−/−^ mice might contain more DSBs, which heightens genomic instability of embryos^43^.

In summary, our study uncovered a novel stem cell-specific pathway *Discn*-NCL-RPA which regulates RPA availability and genomic stability, and plays physiological role in mouse neurogenesis. Our attempts to identify the tissue expression pattern of human *DISCN* using publicly available RNA-seq data were unsuccessful. Future works are needed to investigate the human *DISCN* expression patterns and functions, and its relevance to human diseases.

## Methods

### Mouse embryonic fibroblasts (MEF) preparation and transfection

MEF was isolated from embryos of CD1 mice at embryonic day 13.5 (E13.5) as previously described^44^. Briefly, pregnant mice were euthanized and the embryos were dissected. The fetus head and internal organs were removed. Then the fetus tissue was minced thoroughly and digested with 3 mL of 0.25% trypsin-EDTA at 37 °C for 10 min. Tripsin was neutralized with DMEM (Gibco, 11965) supplemented with 10% FBS (Gibco, 10099141 C). The suspension was transferred to a 50 mL conical tube to allow the undissociated tissues pellet with gravity for 10 min. MEF in supernatant was transferred to 100 mm dishes for further culture. MEF was cultured in DMEM supplemented with 10% fetal bovine serum. To prepare feeder cells for ESC culture, MEF was expanded for three passages and treated with 5 μg/mL mitomycin C (Sigma, M4287) for 4 h. Inactive MEF was frozen in liquid nitrogen. For ectopic expression of *Discn* in MEF, cells were transfected with lipofectamine 2000 according to the manufacturer’s instructions.

### Mouse ESC culture and induced differentiation

Mouse ESCs (derived in our lab) were routinely maintained on inactivated MEF in the following culture medium: Dulbecco’s Modified Eagle Medium/Nutrient Mixture F-12 (DMEM/F12) medium supplemented with 20% Knockout serum replacement (Gibco, 10828028), 1 mM sodium pyruvate (Gibco, 11360070), 2 mM L-glutamine (Sigma, G8540), 1% non-essential amino acids (Gibco, 11140-035), 0.1 mM β-mercaptoethanol (Sigma; M7522), penicillin (100 U/mL)/streptomycin (100 μg/mL) (Gibco, 15140-122) and 1000 units/mL mouse leukemia inhibitory factor (LIF) (Millipore, ESG1107). For ATR inhibition, cells were treated for 4 h with 10 μM VE-821 (Selleck, S8007).

To obtain differentiated cells, mouse ESCs were passaged with 0.05% Tripsin-EDTA (Gibco, 25200072) and seeded onto 0.1% gelatin-coated dishes twice to remove feeders. Culture medium was replaced with differentiation medium: culture medium without LIF and supplemented with 0.5 μM retinoic acid (Sigma, R2625). After one week induction, the differentiated cells were harvested for RNA extraction.

### Neural stem/progenitor cell (NSPC) culture

Mouse NSPC was previously derived from WT mice at postnatal day 5 (P5)^29^. Cells were seeded in 35-mm dishes pre-coated with Laminin (Sigma, L2020) and maintained in complete medium (STEMCELL Technologies, 05702) supplemented with 10 ng/mL basic fibroblast growth factor (bFGF) (STEMCELL Technologies, 02634), 20 ng/mL recombinant human epidermal growth factor (rhEGF) (STEMCELL Technologies, 02633), and 2 μg/mL heparin (STEMCELL Technologies, 07980).

### Generation of *Discn* Knockout mice

The *Discn*^*+/−*^ mouse strain was generated on the C57Bl/6J genetic background by Cyagen Company (https://www.cyagen.com). Briefly, a pair of gRNA located on both sides of mouse *Discn* gene and Cas9 mRNA were co-injected into mouse zygotes to generate targeted knockout offsprings. The gRNA target sequences were CAATTCATCGGATTTCTGAGAGG and ATAGAGGCACAGTAGATTCGGGG. *Discn* knockout was validated by Sanger sequencing of PCR-amplified fragment. Genotying primers, P1, P2-Mut, and P3-WT, were listed in Supplementary Table 1. PCR-based genotyping was performed using genomic DNAs from mouse tails at the following program: 94 °C for 5 min, followed by 35 cycles of denaturation at 94 °C for 15 s, annealing at 64 °C for 30 s, and extension at 68 °C for 36 s.

Mice were housed in accordance to the Animal Care and Use Committee rules and guidelines of the Kunming Institute of Zoology, Chinese Academy of Sciences. Mice were kept under 12 h dark/light cycle, with daylight from 8:00 to 20:00, ambient temperature 20 to 25 °C, and 40 to 70% humidity. All works on mice were carried out in accordance with the guidelines of the Animal Care and Use Committee of the Kunming Institute of Zoology, Chinese Academy of Sciences. All the studies on mice received ethical approval of the Animal Care and Use Committee of the Kunming Institute of Zoology, Chinese Academy of Sciences.

### RNA sequencing and data analyses

Total RNAs were extracted from cultured cells or from mouse brains using Trizol (Tiangen, DP424). The cDNA libraries were constructed using the TruSeq^TM^ RNA Sample Preparation Kit (Illumina) and sequenced using an Illumina HiSeq™ 3000 or HiSeq X Ten platform. To identify novel lncRNAs, the clean reads were mapped to mouse reference genome (mm9) using Tophat2 software (Version 2.0.8). Transcripts were reassembled by Cufflinks (Version 2.1.1)^45^. Non-coding transcript annotation file was downloaded from the NONCODE database (http://www.noncode.org/) (Version 4.0). Novel lncRNAs were identified by Coding-Non-Coding Index (CNCI) software (Version 2)^46^. Differentially expressed lncRNAs were determined by DEGseq software (Version 1.38.0)^47^.

For coding gene analysis, the clean reads were mapped to mouse reference genome (mm10) using Tophat2 software (Version 2.0.8). The values of gene expression were calculated by Cufflinks (Version 2.1.1)^45^. Differentially expressed genes were determined using Cuffdiff software (Version 2.1.1). Gene ontology enrichment was performed using an online tool (http://geneontology.org/)^48,49^. The heatmaps were created by the “gplots” R packages with default parameter. The RNA-seq data have been deposited in the Gene Expression Omnibus (GEO) database (accession number: GSE161998).

### Rapid amplification of cDNA ends (RACE)

The RACE protocol is based on published Smart-seq2 protocol^50^. The oligo-dT primer, template-switching oligos (TSO) and ISPCR primers were synthesized as described in the Smart-seq2 method. For 5ʹ end amplification of *Discn*, reverse transcription was performed using gene specific primer 1 (GSP1). TSO primer was used for template switching. 5ʹ end was amplified by PCR using gene specific primer 1 and ISPCR primer. For 3ʹ end of *Discn*, reverse transcription was performed using oligo-dT primer. 3ʹ end was amplified by PCR using gene specific primer 2 (GSP2) and ISPCR primer. The products were cloned into pEasy-blunt vector (Transgene) and validated by the Sanger sequencing. The primers used for PCR were listed in Supplementary Table 1.

### Expression vector construction

To construct the shRNA expression plasmids, shRNA oligos targeting to *Discn* were designed through an online tool of Ambion siRNA Target Finder. The nucleotide sequences, shRNA-control, shRNA#1, shRNA#2, were listed in Supplementary Table 1. The shRNA oligos were synthesized by Tsingke Company (Beijing, China), annealed and inserted into pLKO.1 plasmid using EcoR I and Age I sites.

For *Discn*-expression plasmids construction, full length *Discn* or predicted open reading frames (ORFs) tagged by FLAG at 3ʹ end were separately amplified using standard PCR procedure and inserted into pCDNA3.1(+) plasmid with the following primers: pCDNA3.1-*Discn*, ORF-1 *Discn*, ORF-2 *Discn*, ORF-3 *Discn*, ORF-4 *Discn*, ORF-5 *Discn*, ORF-6 *Discn*, and ORF-7 *Discn*. For construction of *Discn* or *Discn* mutants inducible expression plasmids, *Discn* or *Discn* mutants were inserted into pTRIPZ vector which carries Tet ON promoter using the following primers: Inducible-*Discn*, *Discn* 5ʹ end, *Discn* 3ʹ end, and *Discn* central part. Expression vectors were packaged into lentivirus for transfection.

pTomo-RPA70-P2A-RPA32-P2A-RPA14 fusion construct was made as followings. Coding sequences for three RPA subunits (RPA70, RPA32, and RPA14) were obtained from cDNA transcribed from mRNA of mESCs by standard PCR procedures with the primers RPA70-p, RPA32-p, and RPA14-p. The RPA fragments were joined with P2A using overlap PCR with the following primers: RPA joint primer, RPA70-P2A primer, P2A-RPA32-P2A primer, and P2A-RPA14-primer. The fragment containing RPA70-P2A-RPA32-P2A-RPA14 was then ligated into pTomo plasmid. The primers used for PCR were listed in Supplementary Table 1.

### Lentivirus package and cell transfection

HEK293T cells were seeded in 15-cm dishes for 24 h, co-transfected with 12 μg expression vector (pLKO.1 or pTRIPZ or pTomo), 6 μg pSPAX, and 6 μg pM2D 2.0 plasmid using polyethyleneimine (PEI) (Sigma). After transfection for 16 h, the medium was replaced by fresh DMEM with 10% FBS. The viral supernatants were collected at 48 h and 72 h post-transfection, respectively. The viruses were filtered through a 0.45 μM syringe filter and concentrated through an Amicon® Ultra-15 Centrifugal Filter (Millipore, R9EA11841).

For *Discn* knockdown or inducible expression, cells were transducted with virus and 8 μg/mL polybrene, and selected with 0.5 μg/mL puromycin.

### Metaphase spread preparation

Cells were treated with 0.02 μg/mL colcemid (Sigma, C3915) for 2 h to arrest in metaphase. Cells were then harvested, re-suspended in hypotonic buffer (0.4% sodium citrate and 0.4% KCL) and incubated for 15 min at 37 °C. After cell swelling, 2 mL freshly prepared methanol-acetic acid fixative (3:1) was added into the hypotonic buffer. Cells were pelleted by centrifuging for 5 min at 200 g. The pellet was re-suspended in 5 mL of 3:1 methanol-acetic acid for 20 min and dropped onto slides to obtain metaphase spreads. At least 50 metaphases were analyzed in three independent replications.

### Sister chromatid exchange (SCE) assay

SCE assay was performed as previously described^6,51^. Briefly, 1 × 10^6^ mouse ESCs were cultured without feeder in ESC medium supplemented with 20 μM bromodeoxyuridine (BrdU, Sigma) for 24 h. In the final 2 h, cells were treated with 0.02 μg/mL colcemid. For SCE assay of mouse NSPCs, cells were cultured in NSPC medium adding 20 μM BrdU for 48 h. In the final 4 h, NSPCs were incubated with 0.02 μg/mL colcemid. Cells were harvested and re-suspended in hypotonic buffer (0.4% sodium citrate and 0.4% KCL) for 15 min at 37 °C. The swollen cells were fixed in freshly prepared methanol-acetic acid fixative (3:1) for 30 min and dropped onto ice-cold wet slides. The slides were dried at 65 °C overnight. The dried slides with metaphase spreads were immersed in 10 μg/mL of Hoeschst 33258 for 20 min and washed with 2 × SSC buffer (0.3 M NaCl, 0.03 M sodium citrate; pH 7.0) once. Then the slides covered with lens paper were exposed coverslip-side-up in 55 °C 2 × SSC buffer to 365 nm UV light at a distance of 1 cm for 30 min. The slides were immersed in 1 × SSC and incubated for 1 h at 50 °C. The slides were stained with 10% Giemsa solution (Gibco, 1892798) for 10 min at 37 °C. After washing with water and dried overnight at room temperature, SCEs images were captured using an Olympus confocal microscopy system.

### Neutral comet assay

The neutral comet assay was performed as previously described^6^. Briefly, cells were dissociated into single cells at the concentration of 1 × 10^5^ cells/mL. Cells were then mixed with low-melting agarose at a ratio of 1:8 and spread onto the slides. Cells were lyzed in lysis buffer (2.5 M NaCl, 100 mM EDTA, 10 mM Tris, 1% N-lauroylsarcosine, and 1% TritonX-100) at room temperature for 1 h. Electrophoresis was performed in electrophoresis buffer (300 mM sodium acetate, 100 mM Tris, pH = 8.3) for 30 min at 80 mA. DNA was fixed in 100% ethanol and stained with DAPI (10 ng/mL) (Thermo). The comet tails were analyzed by Komet 7 comet assay software (Andor Technology). At least 100 cells were analyzed per group. Three independent replications were performed.

### Immunofluorescence staining in cells

For immunofluorescence staining, cells were fixed in 4% paraformaldehyde (PFA), permeabilized in 0.5% Triton X-100, blocked in 2% BSA for 2 h and incubated with primary antibodies at 4 °C overnight. After washing with 0.2% Tweeen 20/PBS three times at room temperature, cells were incubated with secondary antibodies at room temperature for 1 h. Cells were washed three times with PBS and counterstained with DAPI for 10 min. After washing with PBS three times again, slides were sealed with snail oil. Antibody information was listed in Supplementary Table 2.

### Brain section, hematoxylin and eosin (H&E) staining, and immunostaining

After induction of deep anesthesia, mice were transcardially perfused with ice-cold phosphate-buffered saline (PBS) followed with 4% paraformaldehyde (PFA) in PBS. Then the brains were harvested and post-fixed in 4% PFA overnight at 4 °C. After sunk in 15 and 30% sucrose in PBS, the brains were embedded in OCT (optimal cutting temperature compound) for sectioning. H&E staining and immunostaining were performed following the standard protocol.

### Nucleolus and nucleoplasm fractionation

Nucleoli were isolated as previously described^52^. Briefly, 2 × 10^7^ cells were harvested and resuspend in ice-cold hypotonic buffer (10 mM NaCl, 1 mM MgCl_2_, 10 mM Tris.HCl, pH = 7.4) supplemented with Roche complete protease inhibitor (Roche Applied System) and Ribonucleoside Vanadyl Complex (10 mM) (New England BioLabs, S1402S) to have cytoplasm detach from nuclei. Cells were lysed by a 0.45-mm clearance 1-mL Dounce homogenizer. Nuclei were purified and broken by sonication in ice-cold water. Nucleoli were then purified through a sucrose cushion.

Nucleoplasm fractionation was performed as previously described^53^. Briefly, cells were washed once with ice-cold PBS, and then fixed in 1% formaldehyde for 15 min. Cells were lyzed in lysis buffer (25 mM Tris, pH = 7.4, 0.1% Triton X-100, 85 mM KCl). The nuclei were pelleted by centrifuging for 5 min at 2300 × *g*. Then the nuclei were lyzed in SDS buffer (50 mM Tris pH = 7.4, 10 mM EDTA, 4% SDS). After centrifuging at 16,000 × *g* for 30 min, the supernatant was collected as nucleoplasm.

### RNA fluorescence in situ hybridization (FISH) and immunostaining

For RNA-FISH of *Discn*, DNA probes and RNA-FISH hybridization kit were obtained from Guangzhou RiboBio Co., LTD. FISH was performed according to the manufacturer’s protocol with small modification. Briefly, cells were grown on glass covers pre-coated by Matrigel (BD), permeabilized with CSKT buffer [10 mM PIPES (pH = 6.8), 100 mM NaCl, 3 mM MgCl_2_, 0.3 M sucrose and 0.5% Triton X-100] at 4 °C for 10 min and fixed in 4% paraformaldehyde at room temperature for 10 min. Hybridization was performed at 37 °C overnight. Glass covers were washed for 5 min three times with 4 × saline sodium citrate buffer (SSC), once with 2 × SSC, once with 1 × SSC at 42 °C and once with PBS at room temperature. Immunostaining was performed as described in section of immunofluorescence staining for cells.

### Cell proliferation assay

To examine the proliferation, dividing cells were labeled with 10 mM BrdU for 30 min followed by fixation in 4% formaldehyde for 10 min and denaturation in 2 M HCl at room temperature for 1 h. After staining with anti-BrdU primary antibody (Novus) and Cy3 conjugated secondary antibody, the BrdU^+^ cells were sorted by using a FACS LSRFortessa flow cytometer (BD) and BD FACSDiva software (Version 8.0.2). Data were analyzed with Flowjo software (Version 7.6). The gating strategy for BrdU^+^ cells was presented in Supplementary Fig. 8.

### In vitro RNA pulldown and mass spectrometry analysis

RNA was in vitro transcribed with T7 RNA polymerase (Fermentas, EP0111), and was labeled with biotin using Pierce^TM^ RNA 3ʹ End Desthiobiotinylation Kit (Thermo, 20163) according to the manufacturer’s instructions. In vitro RNA pulldown was performed as previously described^54^. Breifly, 50 pmol of labeled RNA in RNA structure buffer (10 mM Tris pH 7, 0.1 M KCl, 10 mM MgCl_2_) was heated to 95 °C for 2 min, stand on ice for 3 min, then left at room temperature (RT) for 30 min to allow proper secondary structure formation. Samples were subjected to RNA pulldown using Pierce^TM^ Magnetic RNA-Protein Pull-Down Kit (Thermo, 20164). Beads were eluted and boiled in 2× SDS protein loading buffer. The recovered proteins were subjected to gradient gel electrophoresis and mass spectrometry (MS) for protein identification or detected by western blot.

### In vivo RNA pulldown

DNA probes for in vivo pulldown were ordered from Guangzhou RiboBio Co., LTD and labeled with biotin at 3ʹ end. In vivo RNA pulldown was performed as previously described^55^. Briefly, 2 × 10^8^ cells were washed with ice-cold PBS once, cross-linked with 265 nm UV light at 400 mJ energy in ice-cold PBS, treated with CSKT buffer supplemented with 1 mM PMSF and SUPERaseIn at 4 °C for 10 min. After centrifuge for 10 min at 1200 × *g*, the pellet was resuspended in 3 mL of DNase I buffer (50 mM Tris pH 7.5, 0.5% Nonidet-P 40, 0.1% sodium lauroyl sarcosine, 1 × protease inhibitors, SUPERaseIn, 600 U RNase free DNase I, 10 mM vanadyl ribonucleoside complex) and incubated at 37 °C to dissolve the pellet followed by a spin. The supernatant was pre-cleared by 50 μL of M-280 streptavidin Dynabeads (Thermo, 00781251) at room temperature for 20 min, and beads were discarded. Pre-cleared lysate, 100 pmol of probes and 160 μL beads were combined, incubated at room temperature for 20 min, preheated to 65 °C for 15 min and followed by slowly cooling to 37 °C for 1 h. The beads were washed 5 min for three times with wash buffer 1 (50 mM Tris, pH 7.5, 0.3 M LiCl, 1% SDS, 0.5% Nonidet-P 40, 1 mM DTT, 1 mM PMSF, 1 × protease cocktail inhibitors (Roche)) at 37 °C, treated with 20 U of DNase I in 300 μL of DNase I buffer at 37 °C for 10 min. Beads were washed with wash buffer 1 twice at 37 °C and buffer 2 (1% SDS, 1 mM DTT, 5 mM EDTA, 150 mM NaCl, 1 mM PMSF) once. Proteins were eluted and boiled in 2 × protein loading buffer at 100 °C for 15 min.

### RNA immunoprecipitation (RIP)

RIP was performed as previously described^56,57^ with minor modifications. Briefly, 2 × 10^7^ cells were lysed in lysis buffer (10 mM HEPES pH 7.0, 100 mM KCl, 5 mM MgCl_2_, 0.5% NP40, 1 mM DTT plus 0.5 U SUPERaseIn (Thermo, AM2694) and proteinase inhibitors) at −80 °C overnight. The lysates were centrifuged at 15,000 × *g* for 15 min at 4 °C. The supernatants were incubated with protein A/G agrose (Abmart, A10001) precoated either with 2.5 μg of normal rabbit IgG antibody (Sigma, I5006) or with 2.5 μg of NCL antibody (Abcam, ab22758) for 4 h at 4 °C. Then beads were washed 5 times with 1 mL of ice-cold NT2 buffer. To release RNA, beads were resuspended in 150 μL of Proteinase K/NT2 buffer (1% SDS, 2.4 μg/μL of Proteinase K) and incubated for 30 min at 55 °C. RNA was extracted with TRNzol Universal (Tiangen, DP424). The reverse transcription of the immunoprecipitated and input RNAs started from the same volume. Fold enrichment was calculated above sample-specific background as described^56^.

### DNA fiber assay

Cells were labeled with 50 μM 5-iodo-2′ -deoxyuridine (IdU; Sigma, I7125) for 30 min and then incubated with 50 μM 5-chloro-2′ -deoxyuridine (CldU; Sigma, C6891) for 30 min, with or without treatment of hydroxyurea (HU, Selleck, S1896). DNA fibers were made as described^7^. Briefly, cells were harvested and suspended in PBS at a concentration of 10^6^/mL. 2,500 cells were lyzed in 12 μL of spreading buffer (0.5% SDS, 50 mM EDTA, 200 mM Tris, pH 7.4) on one end of the glass slide. DNA fibers were spread along the slide by tilting the slides, fixed with freshly prepared methanol-acetic acid (3:1), and treated with 2.5 M hydrochloric acid. For detection of labeled fibers, rat anti-BrdU/CIdU (BU1/75) monoclonal antibody (Novus, NB500-169) and mouse anti-IdU monoclonal antibody (BD, 347580) were used as the primary antibody. The secondary antibodies were AlexaFluor 488-conjugated goat anti-mouse IgG or Cy3-conjugated goat anti-rat IgG. Images were captured from Olympus confocal microscope. The length of labeled fibers were measured using the Image J software, and values were transformed into kilobases using the transform factor 1 μm = 2.59 kb. At least 200 fibers were analyzed for stalled fork restart and nascent DNA degradation.

### New replication origin firing analysis

Origin firing analysis was performed as described^58^. Briefly, cells were pulse-labeled with 50 μM CIdU for 10 min without HU or with 0.1 mM HU for 20 min. DNA fibers including at least four consecutive tracks were selected for measurement. At least 50 DNA fibers were analyzed.

### Quantitative reverse transcription-polymerase chain reaction (qRT-PCR)

Total RNA was isolated using Trizol (Tiangen) protocol. 1 μg of total RNA was reverse transcribed using PrimeScript^TM^ RT Reagent Kit with gDNA Eraser (Takara, RR037A), according to the manufacturer’s protocol. qRT-PCR was performed on CFX96^TM^-Real Time Systerm (Biorad) using the TB Green™ Premix Ex Taq™ II kit (Takara, RR820A). The sequences of the primers used were listed in Supplementary Table 1.

### *Discn* copy number measurement

The cells were counted and lyzed in TRNzol Universal (Tiangen, DP424). The External RNA Controls Consortium (ERCC) RNA Spike-In Control Mixe 1 (Thermo, 4456740) was added into the cell lysate as control. RNAs were isolated for downstream qRT-PCR. ERCC-00042 was used to generate a standard curve for absolute quantification of ERCC-00042 abundance in recovered RNAs. The efficiency of RNA recovery was calculated as the ratio of recovered ERCC-00042 amount to initial ERCC-00042 amount. The copy numbers of *Discn* were determined by comparing Ct values of *Discn* to the Ct values of ERCC-00042, followed by calibration with the RNA recovery efficiency.

### Laser micro-irradiation

Cells were plated on coverslips pre-coated with matrigel. A 405-nm laser of Olympus FV1000 confocal microscope was used for micro-irradiation. Cells were allowed to recover for 2 h. To label S phase of cells, 50 μM BrdU were added in the final 30 min. Then cells were fixed in 4% PFA and subjected for immunostaining. At least 50 cells per experiment were analyzed. Three independent experiments were performed.

### DNA repair assay using HR-RFP and NHEJ-GFP reporter cells

Mouse ESC lines containing NHEJ reporter and HR reporter, and the I-SceI expression plasmid, pcDNA3β-I-SceI were kindly provided by professor An-yong Xie, School of Medicine, Zhejiang University. mESC reporters were transfected with pcDNA3β-I-SceI and GFP or RFP expression plasmid using Neon transfection system. At three days post-transfection, cells were analyzed for RFP^+^ or GFP^+^ frequencies using a FACS LSRFortessa flow cytometer (BD), BD FACSDiva software (Version 8.0.2), and Flowjo software (Version 7.6). The gating strategies for HR-RFP^+^ cells and NHEJ-GFP^+^ cells were presented in Supplementary Fig. 8.

### Immunoprecipitation and western blotting

Cells were lysed in RIPA buffer (Beyotime, P0013J) supplemented with 1 × proteinase inhibitor (Beyotime, P1006). Immunoprecipitation with 2.5 μg of RPA32 antibody or 2.5 μg of isotype IgG (Sigma, I5006) per sample was performed using Protein G Dynabeads (Thermo, 88847) according to the manufacturers’ protocol.

For western blot, total protein was extracted using RIPA buffer (Beyotime, P0013J). Proteins were subjected to 4%-12% SDS-PAGE gels for separation and transferred to polyvinylidene fluoride (PVDF) membrane (Roche, 03010040001). The membrane was blocked with 5% non-fat milk at room temperature for 2 h and then incubated with primary antibodies at 4 °C overnight. After incubation with anti-mouse or rabbit HRP-conjugated secondary antibodies for 1 h at room temperature, the bands were revealed using ECL reagent (Beyotime, P0018FS). Antibodies used were listed in Supplementary Table 2.

### Mouse behavioral tests

All behavioral tests were performed using two months old male mice from littermates. All experiments and data analyses were performed in a blinded manner. The behavioral studies including open-field test, light–dark shuttle box test, Morris water maze test and self-grooming test were performed as previously reported^29^. Briefly, Morris water maze test was carried out in a tank with 120-cm diameter and 50-cm depth. The tank was equipped with a 10-cm diameter platform submerged 1 cm under the water surface masked using white beads. Mice were trained to find the platform within 60 s and stay on the platform for 15 s. Training was terminated once one of the groups succeeded in landing on the platform within 10 s. At 4 and 72 h following the last training, the platform was removed and mice were tested to locate the platform within 60 s in the pool. Mice were video-tracked and their trace was analyzed using the SMART 3.0 software (Panlab Harvard, MA, USA).

Open-field test was performed in an open-top chamber (40 inches by 40 inches by 40 inches). Each mouse was placed in a chamber for 1 h. The activities of mice in the box, including the time spent in the center area and entries to center area, were vedieo-tracked and analyzed by the SMART 3.0 software (Panlab Harvard, MA, USA).

Light–dark box test was performed in an instrument composed of a 18 cm by 27 cm black box and a 27 cm by 27 cm light box. Mice were initially placed into the dark box and allowed to shuttle to the light box for 30 min. The time spent in light box was analyzed.

For self-grooming test, mice were placed in a new cage filled with fresh bedding. Total time spent on grooming and the numbers of grooming bouts were recorded for 10 min.

For rotarod test, the rotation speed of rod was 20 revolutions per minute (rpm) during each trial that lasted for 3 min. Each mouse was tested for three trials with an interval of 30 min. The time spent on the rotating rod was used for evaluation of the motor coordination and balance^59^.

### Statistical analyses

Statistical analyses were performed by two-tailed Student’s *t*-test, two-way ANOVA with Bonferroni post using GraphPad Prism (Version 8.0.1). *P* < 0.05 was considered significant. Statistical details of the experiments were described in the figure legend or in the methods section. *P* values were labeled on the panels. All experiments were performed at least three times.

### Reporting summary

Further information on research design is available in the Nature Research Reporting Summary linked to this article.

## Supplementary information

Supplementary Information

Reporting Summary

## Data Availability

The data that support this study are available from the corresponding author upon reasonable request. The sequence of *Discn* has been deposited in GenBank under accession number MZ269527. RNA-seq data are available in Gene Expression Omnibus database under accession number GSE161998. The mouse reference genome (mm9 and mm10) and annotations can be downloaded through the Illumina’s iGenomes project (http://ccb.jhu.edu/software/tophat/igenomes.shtml). Non-coding transcript annotation file can be downloaded from the NONCODE database (http://www.noncode.org/). For each figure, raw data are provided in the source data files.  Source data are provided with this paper.

## Source data

Source data

## Code availability

The “gplots” code for data visualization (R version 3.6.1) are available from the corresponding author on reasonable request.
