# Supplementary material for: A novel lncRNA Discn fine-tunes replication protein A (RPA) availability to promote genomic stability
Source: Nat Commun. 2021 Sep 22;12:5572. doi: 10.1038/s41467-021-25827-6 (PMC8458541; doi:10.1038/s41467-021-25827-6)
Supplement: Supplementary file 1 — Supplementary Information [file 41467_2021_25827_MOESM1_ESM.pdf]

A novel lncRNA *Discn* fine-tunes replication protein A (RPA) availability to promote genomic stability

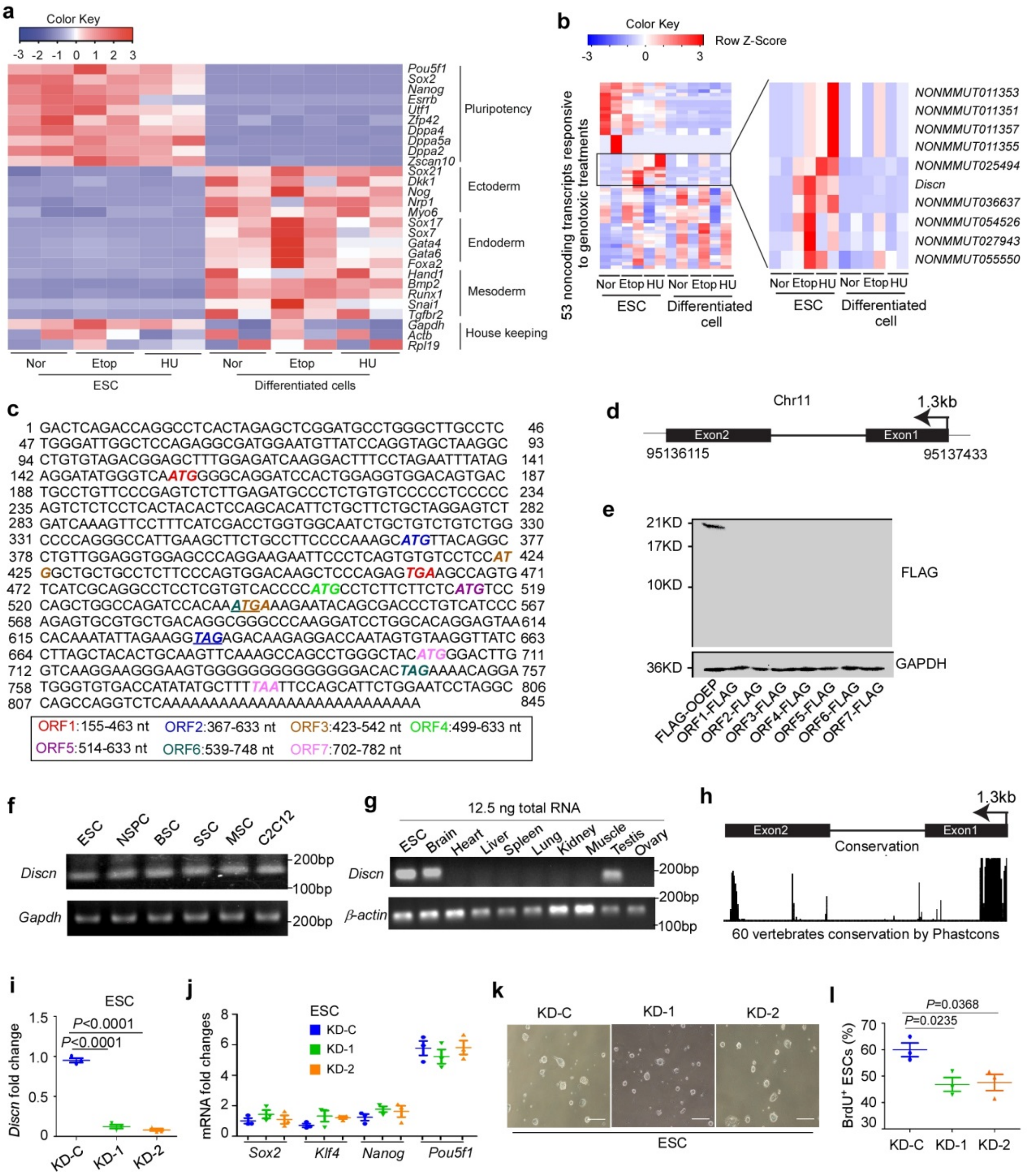

Supplementary Figure 1. Identification and characterization of lncRNA *Discn*.

a Mouse ESCs and differentiated cells were cultured under normal condition (Nor) or treated with 2 mM hydroxyurea (HU) or 10 μM etoposide (Etop) for 4 hours. RNA sequencing verified that the pluripotency genes were highly expressed in ESCs,

whereas the genes monitoring the three germ layer differentiation were highly expressed in differentiated cells. **b** RNA sequencing identified a list of lncRNAs whose expressions were predominantly induced by HU or Etop treatment in ESCs rather than in their differentiated progenies (fold change ≥2). **c** Full length of *Discn* obtained by rapid amplification of cDNA ends (RACE). Predicted open reading frames (ORF) were listed. **d** Chromosome locus of *Discn*. Black boxes indicate exons. Black arrow indicates transcription direction. **e** Transfection experiments failed to detect the expression of any protein or peptide from the coding frames. Flag-tagged OOEP protein was used as positive control. Three independent experiments were repeated with similar results. **f** RT-PCR detected the expression of *Discn* in several tissue stem/progenitor cells including mouse ESC, mouse neural stem/progenitor cell (NSPC), mouse breast stem cell (BSC), mouse spermatogonial stem cell (SSC), mouse mesenchymal stem cell (MSC), and mouse myoblast cell C2C12. Three independent experiments were repeated with similar results. **g** *Discn* expression was restrictively detected in brain and testis by RT-PCR. Three independent experiments were repeated with similar results. **h** Sequence conservation of *Discn* in 60 vertebrates predicted by UCSC Genome Browser track (Phastcons). **i** *Discn* was knocked down (KD) in mESCs by two independent shRNAs (KD-1 and KD-2, respectively). The knockdown efficiency was measured by quantitative RT-PCR. The amount of *Discn* in knockdown control (KD-C) was set as 1.0. Data were shown as mean ± SEM from three replications. Two-tailed Student's t-test. **j** Quantitative RT-PCR analysis of pluripotent transcription factor expressions in mouse ESC with proficient (KD-C for knock down control) or knockdown *Discn* (KD-1 and KD-2). The amount of *Sox2* in KD-C was set as 1.0. *Discn* knockdown did not cause the gene expression change. Data were shown as mean ± SEM from three independent replications. **k** No

morphological change occurred in mouse ESCs after *Discn* KD. Scale bar, 200 μm. **l** Proliferative cells were labeled by BrdU. Fluorescence-activated cell sorting (FACS) analysis revealed that *Discn* KD decreased the proliferation of mouse ESCs. Data were shown as mean ± SEM from three replications. Two-tailed Student's t-test.

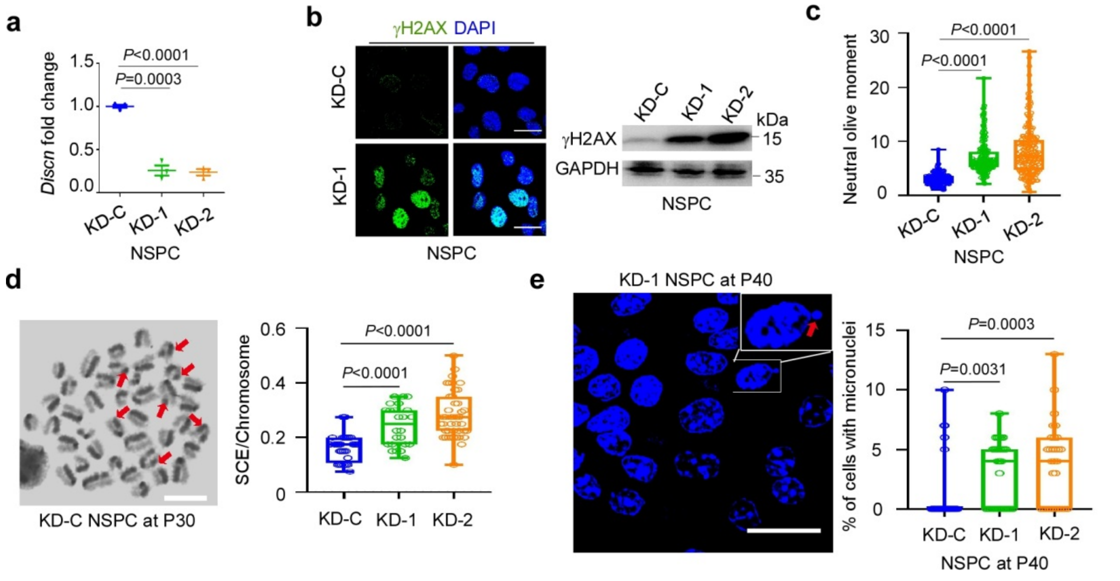

Supplementary Figure 2. Knockdown of *Discn* in mouse NSPC causes genomic instability.

a Quantitative RT-PCR confirmed that *Discn* was efficiently knocked down (KD) by two independent shRNAs in mouse NSPCs. The amount of *Discn* in knockdown control (KD-C) was set as 1.0. $n = 3$ biologically independent samples. Data were shown as mean ± SEM, two-tailed Student's t-test. **b** *Discn* KD caused drastic increase of γH2AX level in cultured NSPCs under normal condition as measured by immunostaining (left panel) and immunoblotting (right panel). Three independent experiments were repeated with similar results. Scale bar, 20 μm. **c** Neutral comet assay validated the increased DNA DSB level in *Discn* KD NSPCs. At least 100 tails were analyzed in each group in three independent replications. **d** Chromosome translocation measured by sister chromatid exchange (SCE) was enhanced in *Discn* KD NSPCs. Left panel showed a representative image of chromosome spread with SCEs (red arrows) and right panel was the quantification. At least 20 metaphase spreads were analyzed in each group. Scale bar, 10 μm. **e** *Discn* KD in NSPCs

increased the micronuclei formation. Left panel showed the representative image of micronucleus (red arrow) and right panel was the quantification. At least 30 visual fields containing 650 cells were analyzed in each cell type. (**c**-**e**) Data were representative of individual values with box and whiskers plots showing the median, upper and lower quartiles, and minimum and maximum. Two-tailed Student's t-test.

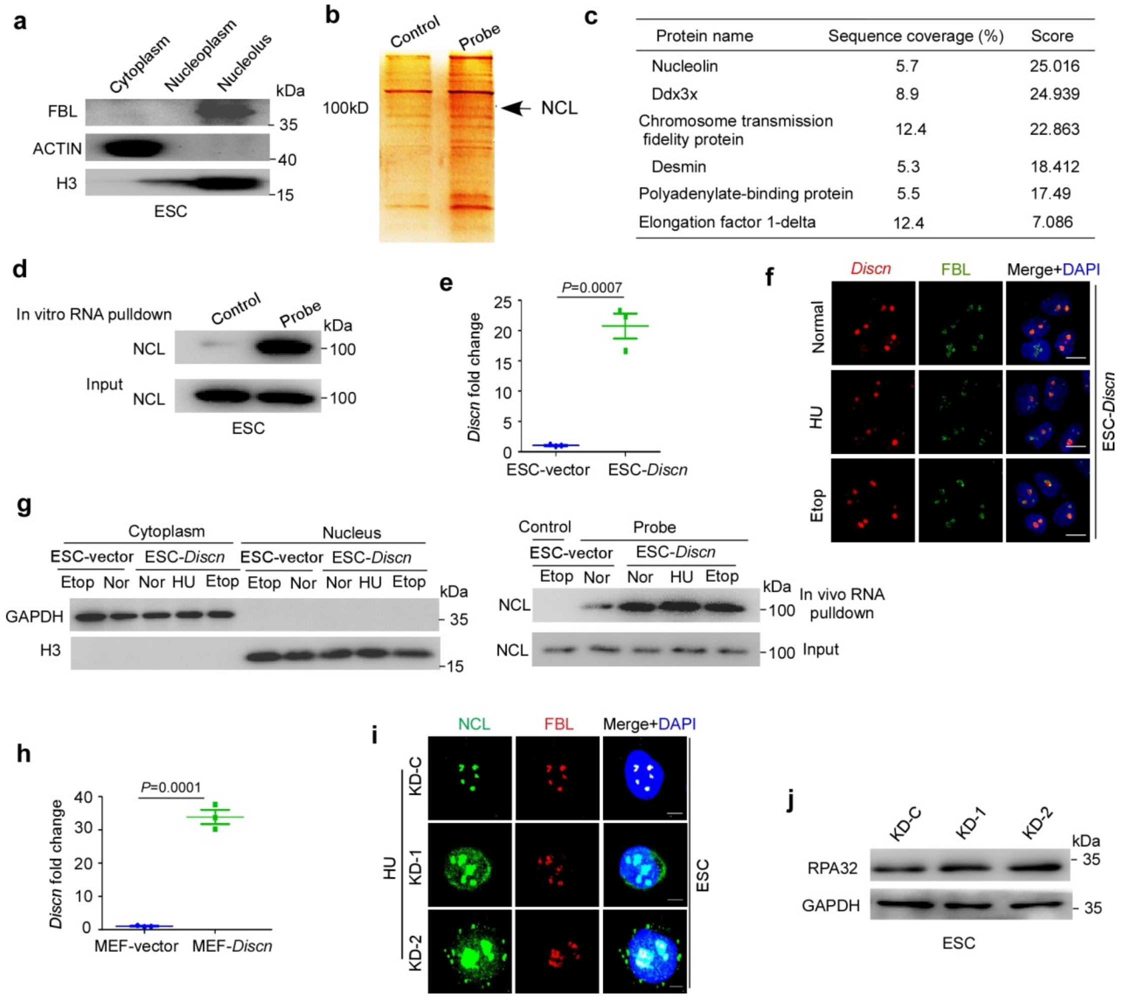

Supplementary Figure 3. *Discn* localizes in nucleolus and interacts with nucleolin (NCL).

a Mouse ESCs were fractionated into cytoplasm, nucleolus and nucleoplasm as indicated by the detection of marker proteins. Three independent experiments were repeated with similar results. **b** *Discn in vitro* RNA pulldown products were separated by SDS-PAGE and stained by silver. Three independent experiments were repeated with similar results. **c** Mass spectrometry analysis identified a list of potential *Discn* binding proteins. Proteins with top high scores were shown here. **d** *In vitro* RNA pulldown combined with immunoblotting confirmed the *in vitro* binding of NCL with *Discn*. Control, antisense strand of *Discn*; Probe, sense strand of *Discn*. Three independent experiments were repeated with similar results. **e** Overexpression of

Disccn in mouse ESCs validated by quantitative RT-PCR. The amount of *Discn* in ESCs transfected with empty vectors (ESC-vector) was set as 1.0. $n = 3$ biologically independent samples, two-tailed unpaired t test. Data were presented as mean ± SEM. **f** In mouse ESCs, over-expressed *Discn* localized in nucleolus labeled with fibrillarin (FBL) no matter whether the cells were cultured under normal, hydroxyurea (HU) or etoposide (Etop) treatment condition. DNA was counterstained with DAPI. Three independent experiments were repeated with similar results. Scale bar, 10 μm. **g** Cells were fractionated into cytoplasmic and nuclear compartments (left panel). In vivo RNA pulldown using nuclear fraction showed that overexpression of *Discn* in mouse ESCs enhanced *Discn*-NCL association under normal culture condition (Nor), and genotoxic treatments (HU and Etop) no longer stimulated the interaction (right panel). Control, sense probe of *Discn*; Probe, antisense probe of *Discn*. Three independent experiments were repeated with similar results. **h** Ectopic expression of *Discn* in MEF as measured by quantitative RT-PCR. The amount of *Discn* in MEFs transfected with empty vectors (MEF-vector) was set as 1.0. $n = 3$ biologically independent samples, two-tailed unpaired t test. Data were presented as mean ± SEM. **i** Immunostaining showed that compared to knockdown control (KD-C), *Discn* knockdown in ESCs (KD-1 and KD-2) promoted NCL translocation to nucleoplasm under 2 mM HU treatment. Scale bar, 5 μm. Three independent experiments were repeated with similar results. **j** Immunoblotting analysis showed that RPA protein expression was not affected by *Discn* knockdown (KD). Three independent experiments were repeated with similar results.

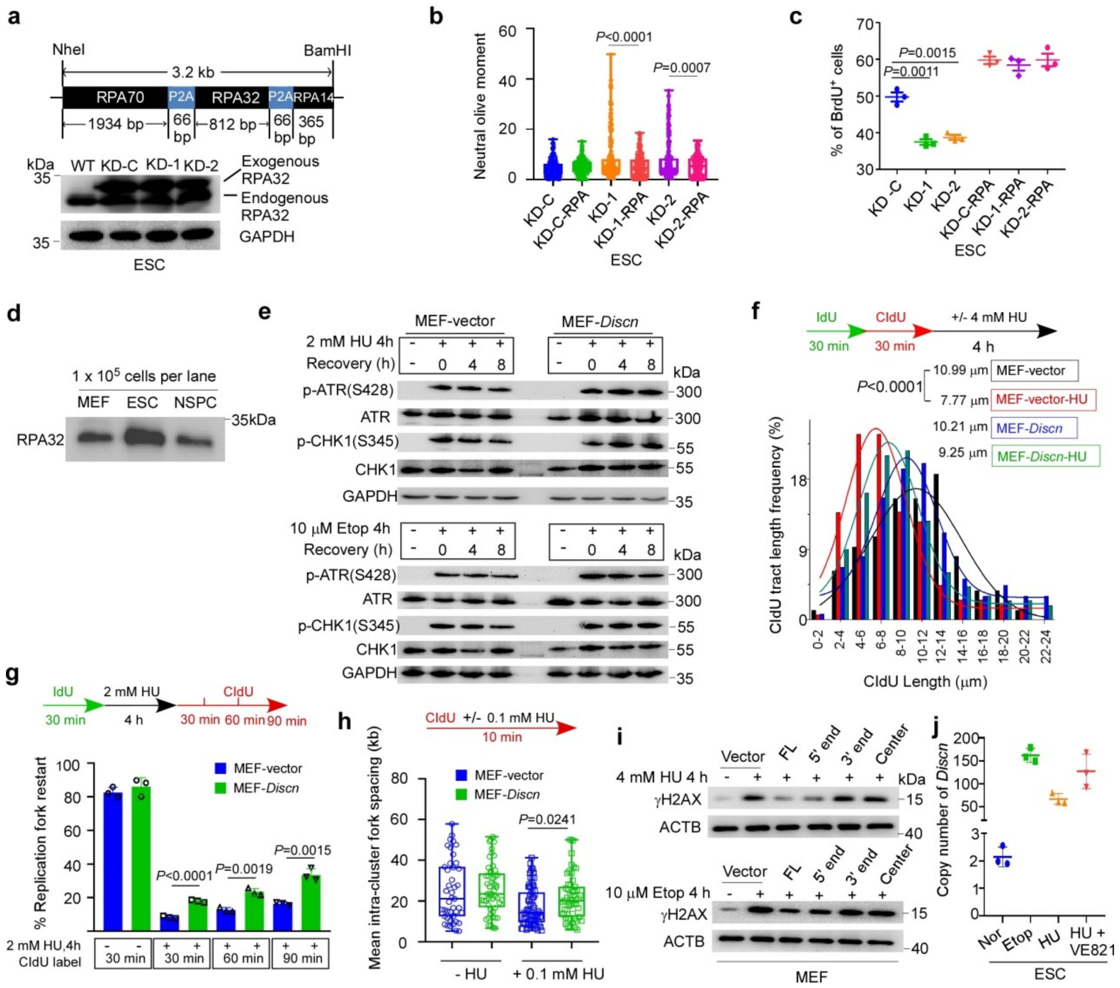

Supplementary Figure 4. RPA mediates the regulatory functions of *Discn* on genomic stability.

a Three subunits of RPA complex (RPA14, RPA32, and RPA70) were co-expressed in *Discn* knockdown control (KD-C) and knockdown ESCs (KD-1 and KD-2). Upper panel depicted the expression construct and lower panel showed the protein expression of RPA. Note that the exogenous RPA was detected in upper band due to the inclusion of P2A fragments. Three independent experiments were repeated with similar results. **b** Neutral comet assay revealed that over-expression of RPA in *Discn* KD ESCs reduced the DNA DSB levels. At least 100 tails were analyzed in each group in three independent replications. **c** RPA overexpression in *Discn* KD ESCs restored their proliferation rate to normal. $n = 3$ biologically independent samples,

two-tailed unpaired t test. Data were presented as mean ± SEM. **d** The RPA protein levels in wild-type MEF, mouse ESCs and NSPCs were compared by immunoblotting. The lysate of 1×10^5 cells were loaded in each well. Three independent experiments were repeated with similar results. **e** Ectopic expression of *Discn* in MEF enhanced the ATR-CHK1 signaling in response to hydroxyurea (HU) or etoposide (Etop) treatment. Three independent experiments were repeated with similar results. Ectopic expression of *Discn* in MEF enhanced their abilities to protect stalled forks from degradation (**f**), to restart stalled forks (**g**), and to suppress dormant origin firing (**h**). At least 200 fibers from three independent replications were analyzed in (**f-g**). At least 50 continuous fibers from three independent replications were analyzed in (**h**). **i** Expression vectors, full length *Discn* (FL), or *Discn* fragments (5′ end, 3′ end, and central part) were introduced into MEFs, respectively. Immunoblotting analyses showed that fragments (3′ end and central part) that do not bind to NCL failed to protect MEFs against genotoxic damages, whereas FL and 5′ end displayed better protective roles. Three independent experiments were repeated with similar results. **j** Average copy numbers of *Discn* per ESC under normal condition (Nor), or treated with 4 h 10 μM etoposide (Etop), 4 h 2 mM hydroxyurea (HU), or 4 h HU plus 10 μM VE-821. $n = 3$ biologically independent samples. In (**b**,**h**), data were representative of individual values with box and whiskers plots showing the median, upper and lower quartiles, and minimum and maximum. Two-tailed Student’s t-test. In (**c**,**f-g**,**j**), data were shown as mean ± SEM, two-tailed Student’s t-test.

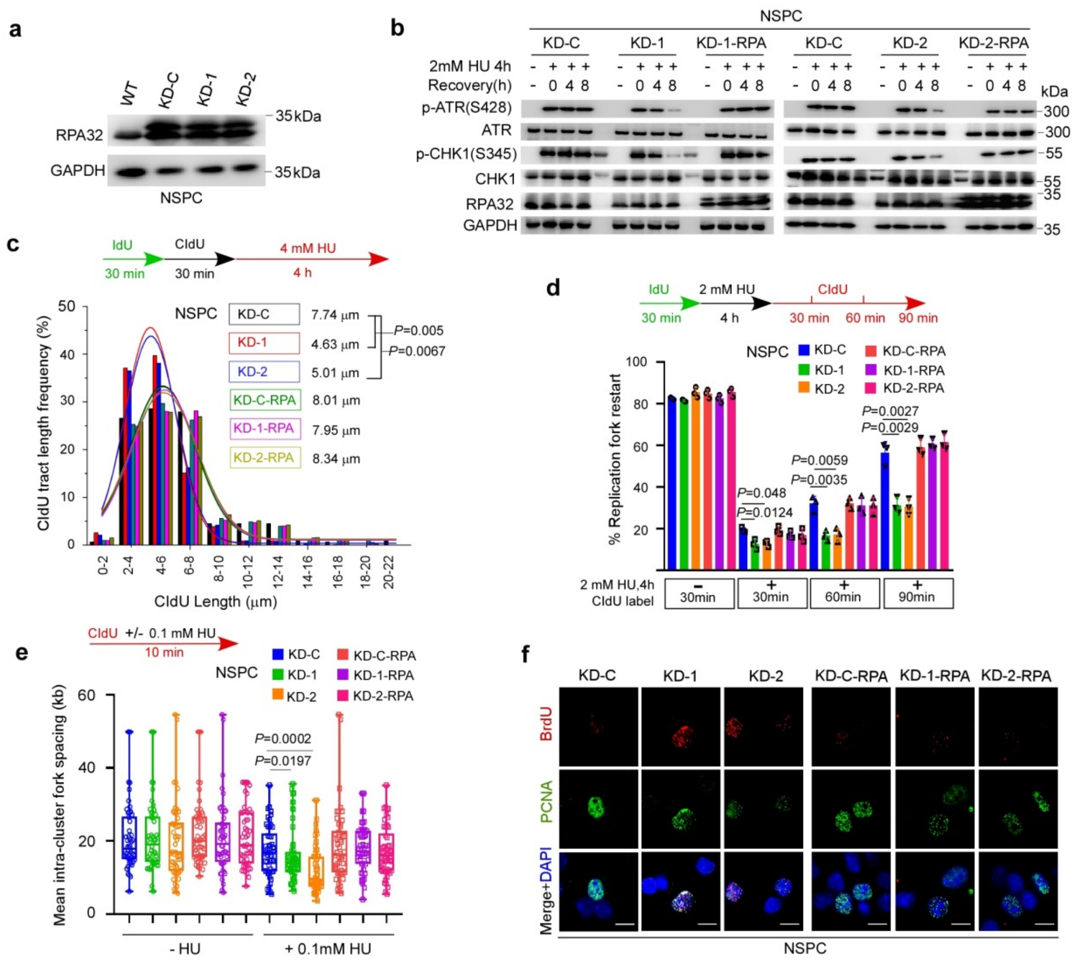

Supplementary Figure 5. Overexpression of RPA rescues the defects of genomic instability in *Discn* KD mouse NSPCs.

a Three RPA subunits as described in supplementary figure 4A were overexpressed in mouse NSPCs. Three independent experiments were repeated with similar results. **b** Overexpression of RPA in *Discn* KD NSPCs (KD-1, KD-2) rescued the ATR-CHK1 signaling. Three independent experiments were repeated with similar results. It also restored the abilities to protect stalled forks from degradation (**c**), to restart stalled forks (**d**), and to suppress dormant origin firing (**e**) as well as ssDNA formation (**f**). Scale bar, 10 μm. (**c-d**) At least 200 fibers from three independent replications were analyzed. Data were shown as mean ± SEM, two-tailed Student's t-test. (**e**) At least 50 continuous fibers from three independent replications were analyzed. Data were

representative of individual values with box and whiskers plots showing the median, upper and lower quartiles, and minimum and maximum. Two-tailed Student’s t-test.

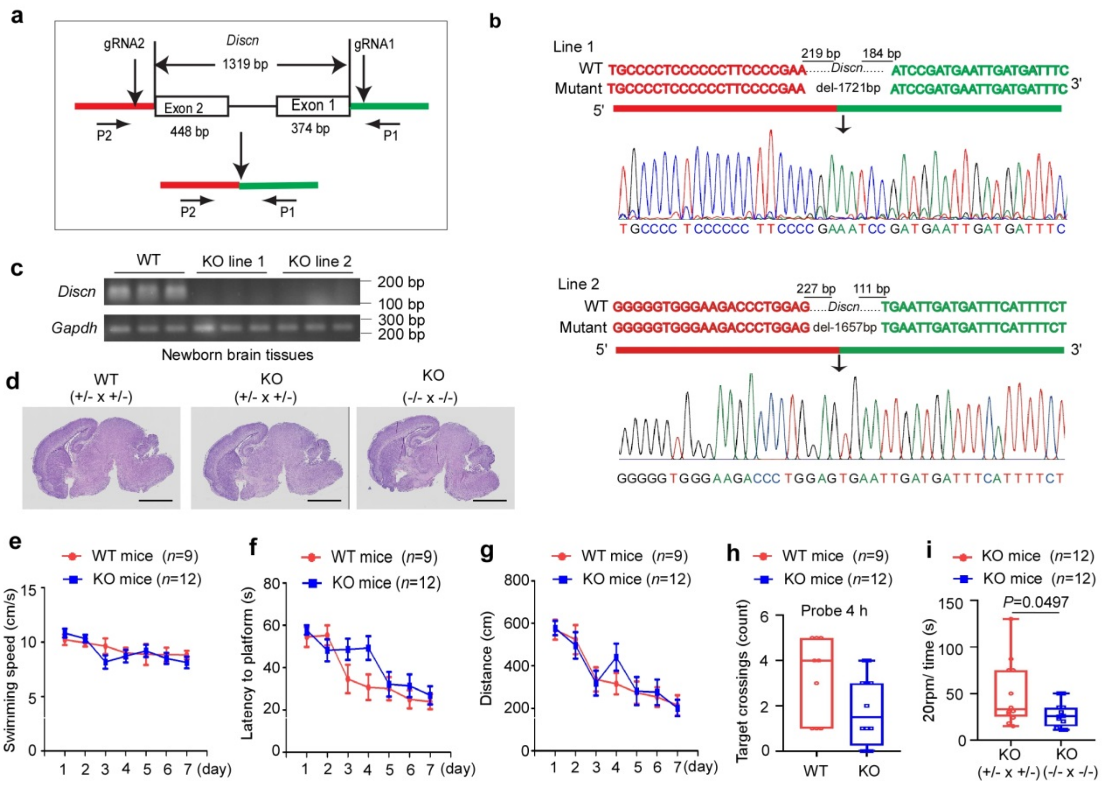

Supplementary Figure 6. Generation of *Discn* knock out (KO) mice and behavioral tests.

a Schematic diagram of CRISPR/Cas9 design targeting the mice *Discn* locus. P1 and P2 are primers for genotyping. **b** *Discn* KO was validated by Sanger sequencing of PCR-amplified fragment. A 1721-bp fragment was deleted in line 1 and a 1657-bp fragment was deleted in line 2. **c** RT-PCR showed the absence of *Discn* expression in KO newborn mouse brains. Three independent experiments were repeated with similar results. **d** Representative images of mouse brains (Hematoxylin and Eosin staining) for WT and *Discn* KO mice. +/- × +/-, mice from *Discn*$^{+/-}$ crossing with *Discn*$^{+/-}$. -/- × -/-, mice from *Discn*$^{-/-}$ crossing with *Discn*$^{-/-}$. At least 5 mice were examined in each group. Scar bar, 2.5 mm. (**e-h**) *Discn* KO and WT mice were littermates from *Discn*$^{+/-}$ crossings. Morris water maze test showed that KO and WT littermates swam in similar speed (**e**). They used similar time (**f**) and traveled similar distance (**g**) in locating the platform during the 7 days of training. **h** At 4 h following

7-day trial, KO and WT littermates showed similar platform crossings. **i** In rotarod test, KO mice from *Discn*$^{+/-}$ crossings stayed on the rotating rod for longer time than KO mice from *Discn*$^{-/-}$ crossings. (**e**-**g**) Data were represented as mean ± SEM. Two-way ANOVA with Bonferroni post hoc test. (**h**-**i**) Data were representative of individual values with box and whiskers plots showing the median, upper and lower quartiles, and minimum and maximum. Two-tailed Student's test.

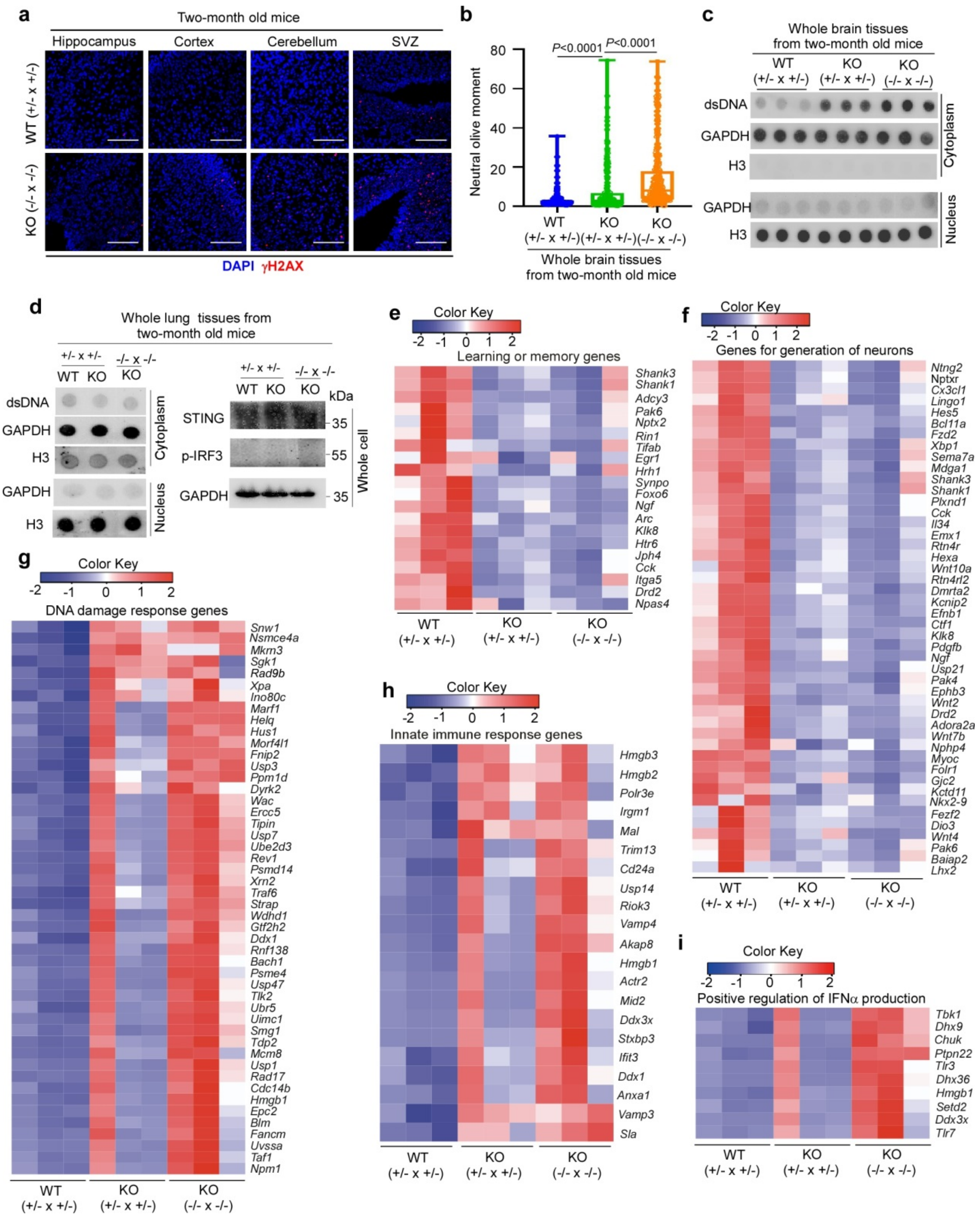

Supplementary Figure 7. Brain cells of adult *Discn* KO mice have elevated DNA damages and severe immune reactions.

a Representative images showed more γH2AX foci in hippocampus, cortex, cerebellum and subventricular zone (SVZ) of adult (2 months old) *Discn* KO mouse brains when compared to the WT counterparts. Scale bar, 100 µm. Three independent experiments were repeated with similar results. **b** Neutral comet assay also revealed

the increased DNA DSB levels in adult *Discn* KO mouse brain cells. Note that KO from *Discn*$^{-/-}$ crossings displayed the most severe damage. At least 100 tails were analyzed in each group in three independent replications. Data were representative of individual values with box and whiskers plots showing the median, upper and lower quartiles, and minimum and maximum. Two-tailed Student's t-test. **c** Adult brain cells were fractionated into cytoplasm and nucleus compartments. Dot immunoblotting showed that brain cells from *Discn* KO mice contained higher level of cytoplasmic double-strand DNA (dsDNA) than those from WT controls. Three independent experiments were repeated with similar results. **d** Adult lung cells were fractionated into cytoplasm and nucleus compartments. Dot immunoblotting showed that there was no significant difference in the cytoplasmic double-strand DNA (dsDNA) between WT and *Discn* KO mice (left panel). Whole lung tissues were subject to western blot. The result showed that the STING pathway was not activated in WT and *Discn* KO mice (right panel). Three independent experiments were repeated with similar results. Heatmaps of differentially expressed genes (fold change ≥ 2, $p < 0.05$, $q < 0.05$) involved in learning and memory (**e**), generation of neurons (**f**), DNA damage responses (**g**), innate immune response (**h**), and positive regulation of IFNα production (**i**) in adult brain cells of three mouse groups. +/- × +/-, mice from *Discn*$^{+/-}$ crossing with *Discn*$^{+/-}$. -/- × -/-, mice from *Discn*$^{-/-}$ crossing with *Discn*$^{-/-}$.

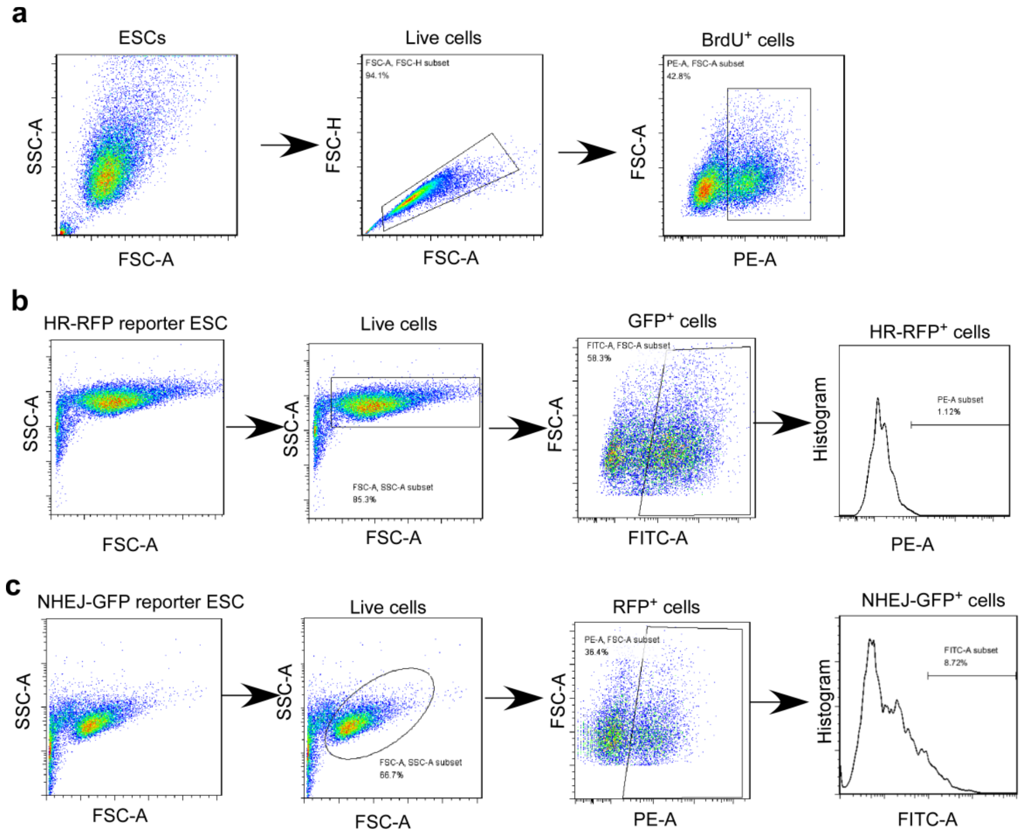

Supplementary Figure 8. The gating strategies for sorting BrdU$^+$ cells and DNA repair reporter cells.

a Gating strategy to determine the BrdU$^+$ cells. **b** Gating strategy to determine the cells undertaking homologous recombination (HR)-mediated DNA repair. **c** Gating strategy to determine the cells undertaking DNA repair via nonhomologous end-joining (NHEJ) pathway.

Supplementary Table 1. Oligonucleotides used in this study.

Gene		Oligonucleotides
Discn shRNA sequences		
shRNA-control	Forward	5'-CCGGTAAGGCTATGAAGAGATACCTCGAGGTATCTCTTCATAGCCTTATTTTTG-3'
	Reverse	5'-AATTCAAAAATAAGGCTATGAAGAGATACCTCGAGGTATCTCTTCATAGCCTTA-3'
shRNA#1	Forward	5'-CCGGGCTAGGAGTCTGATCAAAGTTCTCGAGAACTTTGATCAGACTCCTAGCTTTTTG -3'
	Reverse	5′-AATTCAAAAAGCTAGGAGTCTGATCAAAGTTCTCGAGAACTTTGATCAGACTCCTAGC-3'
shRNA#2	Forward	5′-CCGGGCACAGGAGTAACACAAATATCTCGAGATATTTGTGTTACTCCTGTGCTTTTTG -3'
	Reverse	5′-AATTCAAAAAGCACAGGAGTAACACAAATATCTCGAGATATTTGTGTTACTCCTGTGC -3'
RT-PCR primers		
Gapdh	Forward	5′-CACATTGGGGGTAGGAACAC-3'
	Reverse	5′-AACTTTGGCATTGTGGAAGG-3'
β-actin	Forward	5′- TTGAGGTCAATGAAGGGGTC-3'
	Reverse	5′- TCGTCCCGTAGACAAAATGG-3'
18S rRNA	Forward	5'- GAGGGACAAGTGGCGTTCAG -3'
	Reverse	5′- CATCACGAATGGGGTTCAAC -3'
Discn	Forward	5′- GTGGCAATCTGCTGTCTGTCT -3'
	Reverse	5′- CGATGACACTGGCTTCACTCT -3'
Pou5f1	Forward	5′-GGAGGAAGCCGACAACAA-3′
	Reverse	5′-GGGCAGAGGAAAGGATACAG-3′
Nanog	Forward	5′-GCCCTGATTCTTCTACCA-3′
	Reverse	5′-AGATGCGTTCACCAGATAG-3′

Sox2	Forward	5′-AAACCGTGATGCCGACTA-3′
	Reverse	5′-ATCCGAATAAACTCCTTCCTTG-3′
Klf4	Forward	5′-CCAGAGGAGCCCAAGCCAAAG-3′
	Reverse	5′-CGGTAGTGCCTGGTCAGTTCAT-3′
IFN-α	Forward	5′-CCAGCAGATCAAGAAGGCTCAA-3′
	Reverse	5′-GAAGACAGGGCTCTCCAGAC-3′
IFN-β	Forward	5′-CTGCCTTTGCCATCCAAGAGAT-3′
	Reverse	5′-TTTGCACCCTCCAGTAATAGCT-3′
ERCC-000 42	Forward	5′-GTGGTCTGCATAAGGGTAGAGAG-3′
	Reverse	5′-GCTTTGTCTTTAAACGCTCACCT-3′
RACE primers		
Discn RACE	GSP2	5′-TGGGATTGTATCCAGAGAGTCAAT-3'
	GSP1	5′-TCTGGGGAGGTCCTTGATCTC-3'
Predicted ORFs vector construction primer		
ORF1-*Discn*	Forward	5′-ACTAGCTAGCATGGGGCAGGATCCACTGGA-3'
	Reverse	5′-CCGCTCGAGTCACTTGTCATCGTCGTCCTTGTAGTCCTCTGGGAGCTTGTCCACTGG-3'
ORF2-*Discn*	Forward	5′-ACTAGCTAGCATGTTACAGGCCTGTTGGAGG-3'
	Reverse	5′-CCGCTCGAGTCACTTGTCATCGTCGTCCTTGTAGTCCCTTCTAATATTTGTGTTACTCCT-3'
ORF3-*Discn*	Forward	5′-ACTAGCTAGCATGGCTGCTGCCTCTTCCCA-3'
	Reverse	5′-CCGCTCGAGTCACTTGTCATCGTCGTCCTTGTAGTCTTTGTGGATCTGGCCAGCTGG-3'
ORF4-*Discn*	Forward	5′-ACTAGCTAGCATGCCTCTTCTTCTCATGTCC-3'
	Reverse	5′-CCGCTCGAGTCACTTGTCATCGTCGTCCTTGTAGTCCCTTCTAATATTTGTGTTACTCCT-3'

ORF5-*Discn*	Forward	5′-ACTAGCTAGCATGTCCCAGCTGGCCAGATCC-3'
	Reverse	5′-CCGCTCGAGTCACTTGTCATCGTCGTCCTTGTAGTCCCTT CTAATATTTGTGTTACTCCT-3'
ORF6-*Discn*	Forward	5′-ACTAGCTAGCATGAAAGAATACAGCGACCCTG-3'
	Reverse	5′-CCGCTCGAGTCACTTGTCATCGTCGTCCTTGTAGTCGTGT CCCCCCCCCCCCCC-3'
ORF7-*Discn*	Forward	5′-ACTAGCTAGCATGGGACTTGGTCAAGGAAGG-3'
	Reverse	5′-CCGCTCGAGTCACTTGTCATCGTCGTCCTTGTAGTCAAA GCATATATGGTCACACCCAT-3'
Discn expression vector construction primer		
Inducible-*Discn*	Forward	5′-GCACCGGTGACTCAGACCAGGCCTCACTA-3'
	Reverse	5′-CATCTCGAGACTATAGGGCGAATTGAAGCTG-3'
pCDNA3.1 - *Discn*	Forward	5′-ACTAGCTAGCGACTCAGACCAGGCCTCACTA-3'
	Reverse	5′-CAACTCGAGCTATAGGGCGAATTGAAGCTG-3'
Discn 5′ end	Forward	5′- GCACCGGTACTCAGACCAGGCCTCACTAGA-3'
	Reverse	5′- CATCTCGAGATTGACCCATATCCTCTATAAATTC-3'
Discn 3′ end	Forward	5′-GCACCGGTGAAGGTAGAGACAAGAGGACCAA-3'
	Reverse	5′-CATCTCGAGTTTTTGAGACCTGGCTGGCCT-3'
Discn Central part	Forward	5′-GCACCGGTGGGGCAGGATCCACTGGAGG-3'
	Reverse	5′-CATCTCGAGCCTTCTAATATTTGTGTTACTCCTG-3'
RPA expression vector construction primers		
RPA70-p	Forward	5′-GTGGGACACCTGAGCGAGG-3'
	Reverse	5′-CATGTTCTTCCTGATGTTCGC-3'
RPA32-p	Forward	5′-GGATGTGGAATAGCGGATTCGA-3'

	Reverse	5′-CCCAGGAAACTCCAGTCACTCT-3'
RPA14-p	Forward	5′-ATGGAGGACATAATGCAGCTCC-3'
	Reverse	5′-GAAACTGAAGAAGCTCATTCATGT-3'
RPA joint primer	Forward	5′-CTAGCTAGCGCCACCATGGTGGGACACCTGAGCGAGG-3'
	Reverse	5′-CGGGATCCTCATTCATGTTGTGGAAGCCCTA-3'
RPA70-P2A primer	Reverse	5′-GCTGAAGTTAGTAGCTCCGCTTCCCATGTTCTTCCTGATGTTCGC-3'
P2A-RPA32-P2A primer	Forward	5′-GGAAGCGGAGCTACTAACTTCAGCCTGCTGAAGCAGGCTGGAGACGTGGAGGAGAACCCTGGACCTTGGAATAGCGGATTCGAAAG-3'
	Reverse	5′-TGAAGTTAGTAGCTCCGCTTCCCTCTGCATCTGTAGACTTAAAGT-3'
P2A-RPA14- primer	Forward	5′-GGAAGCGGAGCTACTAACTTCAGCCTGCTGAAGCAGGCTGGAGACGTGGAGGAGAACCCTGGACCTGAGGACATAATGCAGCTCCCC-3'

Mouse genotyping

Discn	P1	5′- CCCCATCACTCCTCTGTACTATT -3′
	P2-Mut	5′- GAGCTGGGGATGGGAGTTCTA -3′
	P3-WT	5’- TTGATCTCCAAAGCTCCGTCTAC -3′

Supplementary Table 2. Antibody information.

**Name**	**Company**	**Catalog#**	**Clone #**	**Dilution**
Rabbit IgG	Sigma	I5006		IP:2.5 μg/sample RIP:2.5 μg/sample
Mouse monoclonal anti-IdU	BD Biosciences	347580	B44	1:500 (IF)
Rat monoclonal anti-BrdU, also used for immunostaining of CIdU	Novus	NB500-169	BU1/75	1:1000 (IF)
p-ATR (Ser428) rabbit antibody	Cell Signaling Technology	2853S		1:1000 (IB)
ATR rabbit polyclonal antibody	Cell Signaling Technology	2790S		1:1000 (IB)
CHK1 mouse monoclonal antibody	Cell Signaling Technology	2360S	261D5	1:1000 (IB)
P-CHK1(S345) rabbit monoclonal antibody	Cell Signaling Technology	2348S	133D3	1:1000 (IB)
ACTB mouse antibody	abclonal	AC004		1:10000 (IB)
GAPDH mouse monoclonal antibody	Sangon Biotech	D190090		1:1000 (IB)
γ-H2AX rabbit polyclonal antibody	Cell Signaling Technology	9718	20E3	1:1000 (IF& IB)
NCL rabbit antibody	Abcam	ab129200	EPR7952	1:1000 (IF, IB)
NCL rabbit antibody	Abcam	ab22758		2.5 μg (RIP)
FBL mouse antibody	Abcam	ab4566	38F3	1:1000 (IF, IB)
RAD51 mouse antibody	Abnova	H00005888-B01P		1:500 (IF, IB)
Histone H3 rabbit antibody	Wanlei	WL02243		1:1000 (IB)
PCNA mouse antibody	Cell Signaling Technology	2586S	PC10	1:1000 (IF)
RPA32 rabbit antibody	Abcam	ab76420		1:10000 (IB) 1 μg/sample
dsDNA marker mouse antibody	Santa cruze	SC-58749		1:500 (IB)
Cleaved mouse caspase 1 (p20) antibody	Adipogen	AG-20B-0042		1:1000 (IB)

IL-1 beta rabbit antibody	Abcam	ab82558		1:1000 (IB)
TMEM173 / STING rabbit antibody	Lifespan Bioscience	LS-B7237		1:1000 (IB)
TBK1/NAK rabbit antibody	Cell Signaling Technology	38066	E8I3G	1:1000 (IB)
Phospho-TBK1/NAK (Ser172) rabbit antibody	Cell Signaling Technology	5483S	D52C2	1:1000 (IB)
IRF-3 rabbit antibody	Cell Signaling Technology	4302S	D83B9	1:1000 (IB)
Phospho-IRF-3 (Ser396) rabbit antibody	Cell Signaling Technology	29047S	D6O1 M	1:1000 (IB)
Goat anti-rat IgG secondary antibody, Alexa Fluor Cy3	Thermo Fisher Scientific	A10522		1:500 (IF)
Goat anti-mouse IgG secondary antibody, Alexa Fluor 488	Thermo Fisher Scientific	A11029		1:500 (IF)
Goat anti-rabbit IgG secondary antibody, Alexa Fluor 647	Thermo Fisher Scientific	A27040		1:500 (IF)
Goat anti-rabbit IgG secondary antibody, Alexa Fluor 488	Thermo Fisher Scientific	A11034		1:500 (IF)
Donkey anti-rabbit IgG secondary antibody, Alexa Fluor 555	Thermo Fisher Scientific	A31572		1:500 (IF)
Goat anti-mouse IgG (H+L) secondary antibody, HRP	Thermo Fisher Scientific	31430		1:5000 (IB)
Goat anti-rabbit IgG (H+L) secondary antibody, HRP	Thermo Fisher Scientific	31460		1:5000 (IB)
