# Supplementary material for: A novel lncRNA Discn fine-tunes replication protein A (RPA) availability to promote genomic stability
Source: Nat Commun. 2021 Sep 22;12:5572. doi: 10.1038/s41467-021-25827-6 (PMC8458541; doi:10.1038/s41467-021-25827-6)
Supplement: Supplementary file 2 — Reporting Summary [file 41467_2021_25827_MOESM2_ESM.pdf]

nature research

Corresponding author(s): Ping Zheng
Last updated by author(s): Jul 29, 2021

Reporting Summary

Nature Research wishes to improve the reproducibility of the work that we publish. This form provides structure for consistency and transparency in reporting. For further information on Nature Research policies, see our Editorial Policies and the Editorial Policy Checklist.

Statistics

For all statistical analyses, confirm that the following items are present in the figure legend, table legend, main text, or Methods section.

n/a	Confirmed	
☐	☒	The exact sample size (*n*) for each experimental group/condition, given as a discrete number and unit of measurement
☐	☒	A statement on whether measurements were taken from distinct samples or whether the same sample was measured repeatedly
☐	☒	The statistical test(s) used AND whether they are one- or two-sided<br>*Only common tests should be described solely by name; describe more complex techniques in the Methods section.*
☒	☐	A description of all covariates tested
☐	☒	A description of any assumptions or corrections, such as tests of normality and adjustment for multiple comparisons
☐	☒	A full description of the statistical parameters including central tendency (e.g. means) or other basic estimates (e.g. regression coefficient) AND variation (e.g. standard deviation) or associated estimates of uncertainty (e.g. confidence intervals)
☐	☒	For null hypothesis testing, the test statistic (e.g. *F*, *t*, *r*) with confidence intervals, effect sizes, degrees of freedom and *P* value noted<br>*Give P values as exact values whenever suitable.*
☒	☐	For Bayesian analysis, information on the choice of priors and Markov chain Monte Carlo settings
☒	☐	For hierarchical and complex designs, identification of the appropriate level for tests and full reporting of outcomes
☒	☐	Estimates of effect sizes (e.g. Cohen's *d*, Pearson's *r*), indicating how they were calculated

Our web collection on statistics for biologists contains articles on many of the points above.

Software and code

Policy information about availability of computer code

Data collection

RNA-seq data: Illumina HiSeq™ 3000 or HiSeq X Ten platform.
FACS: LSRFortessa flow cytometer (BD)
shRNA design: Ambion siRNA Target Finder
qRT-PCR:CFX96TM-Real Time Systerm (Biorad)
Microscope:FV1000 confocal laser scanning biological microscope(Olympus)
Rota-rod task:Rota-rod apparatus (Panlab, LE8500)
Morris water maze behavioural study:a tank with 120-cm diameter and 50-cm deep tank . The tank was equipped with a 10-cm diameter platform submerged 1 cm under the water surface.
Open-field behavioral assessment: An open-top chamber(40 × 40 × 40 inches).
Light-dark box test: an instrument composed of a 18 cm by 27 cm black box and a 27 cm by 27 cm light box.
RNA motif prediction: RegRNA 2.0 online tools (http://regrna2.mbc.nctu.edu.tw/index.html)

Data analysis

Statistics were performed using GraphPad Prism version 8.0.1. To identify novel lncRNAs, the RNA-seq data were mapped to mouse reference genome (mm9) using Tophat2 software (Version 2.0.8). Transcripts were reassembled by Cufflinks (Version 2.1.1). Novel lncRNAs were identified by Coding-Non-Coding Index (CNCI) software (Version 2). Differentially expressed lncRNAs were determined by DEGseq software (Version 1.38.0). For coding gene analysis, the RNA-seq data were mapped to mouse reference genome (mm10) using Tophat2 software with default parameter. The values of gene expression were calculated by Cufflinks. Differentially expressed genes were determined using Cuffdiff software (Version 2.1.1) with default parameter. Gene ontology enrichment was performed using an online tool (http://geneontology.org/). The heatmaps were created by the 'gplots' R packages with default parameter. Flow Cytometry data were acquired using BD FACSDiva software (Version 8.0.2), then analyzed with Flowjo software version 7.6. Morris water maze and Open-field behavioral assessment were analyzed using the SMART3.0 software. Q-PCR results were analyzed using CFX Manager software version 3.1. The comet tails were analyzed by Komet 7 comet assay software. The length of labeled fibers were measured using the Image J software (Version 1.38). RNA motif was

predicted using RegRNA 2.0 online tools (http://regrna2.mbc.nctu.edu.tw/index.html).

For manuscripts utilizing custom algorithms or software that are central to the research but not yet described in published literature, software must be made available to editors and reviewers. We strongly encourage code deposition in a community repository (e.g. GitHub). See the Nature Research guidelines for submitting code & software for further information.

Data

Policy information about availability of data

All manuscripts must include a data availability statement. This statement should provide the following information, where applicable:
- Accession codes, unique identifiers, or web links for publicly available datasets
- A list of figures that have associated raw data
- A description of any restrictions on data availability

Authors confirm that all relevant data are included in the paper and/or its supplementary information files. RNA-seq data are available in Gene Expression Omnibus database under accession number GSE161998 (https://www.ncbi.nlm.nih.gov/geo/query/acc.cgi?acc=GSE161998). The sequence information of Discn have been deposited in GenBank under accession number MZ269527(https://www.ncbi.nlm.nih.gov/nuccore/MZ269527.1/). Source data have been provided. The mouse reference genome (mm9 and mm10) and annotations can be downloaded through the Illumina's iGenomes project (http://ccb.jhu.edu/software/tophat/igenomes.shtml). Non-coding transcript annotation file can be downloaded from the NONCODE database (http://www.noncode.org/).

Field-specific reporting

Please select the one below that is the best fit for your research. If you are not sure, read the appropriate sections before making your selection.

☒ Life sciences ☐ Behavioural & social sciences ☐ Ecological, evolutionary & environmental sciences

For a reference copy of the document with all sections, see nature.com/documents/nr-reporting-summary-flat.pdf

Life sciences study design

All studies must disclose on these points even when the disclosure is negative.

Sample size	Statistical methods were not used to determine sample size. For karyotype assay, fiber assay, neutral comet assay, micro-nuclei assay, and laser micro-irradiation, sample sizes were based on prior experience in the field (Methods Mol Biol. 2014 1105:439-55; Stem Cell Reports. 2015 Aug 11;5(2):185-94.; Cell Res. 2018 Jan;28(1):69-89. Sci Adv. 2020 Oct 28;6(44):eaba0682.). For in vitro experiments, at least three biological replicates were performed for all the experiments. For behavior experiments, the sample sizes were chosen according to the previous works (Nature, 2020, 580(7805):647-652.; Sci Adv, 2020, 6: eaba0682), in which n ≧ 7 mice were used per experimental group.
Data exclusions	No data was excluded from analysis.
Replication	All assays were repeated independently for at least three times. One representative result for each experiment is presented in the figures.
Randomization	Randomization was not relevant because all samples used for analysis were from the same initial stocks.
Blinding	Mouse behavioral tests and data analyses were performed in a blinded manner. Other experiments were not relevant to blinding because they are quantitative and not qualitative in nature.

Reporting for specific materials, systems and methods

We require information from authors about some types of materials, experimental systems and methods used in many studies. Here, indicate whether each material, system or method listed is relevant to your study. If you are not sure if a list item applies to your research, read the appropriate section before selecting a response.

Materials & experimental systems

n/a	Involved in the study	
☐	☒	Antibodies
☐	☒	Eukaryotic cell lines
☒	☐	Palaeontology and archaeology
☐	☒	Animals and other organisms
☒	☐	Human research participants
☒	☐	Clinical data
☒	☐	Dual use research of concern

Methods

n/a	Involved in the study	
☒	☐	ChIP-seq
☐	☒	Flow cytometry
☒	☐	MRI-based neuroimaging

Antibodies

Antibodies used	Rabbit IgG (sigma;Cat#I5006;Application:IP, RIP)

Antibodies used

Mouse monoclonal anti-IdU (BD Biosciences; Cat#347580; Clone#B44;Application:IF)
Rat monoclonal anti-BrdU (Novus; Cat#NB500-169; Clone#BU1/75; Application:IF,FACS)
p-ATR (Ser428) Rabbit antibody (Cell Signaling Technology; Cat#2853S; Application:IB)
ATR Rabbit polyclonal antibody (Cell Signaling Technology; Cat#2790S; Application:IB)
Chk1 Mouse monoclonal antibody (Cell Signaling Technology; Cat#2360S; Clone#261D5; Application:IB)
P-Chk1(S345) Rabbit monoclonal antibody (Cell Signaling Technology; Cat#2348S; Clone#133D3; Application:IB)
Actb mouse antibody (Abclonal; Cat#AC004; Application:IB)
Gapdh Mouse monoclonal antibody (Sangon Biotech; Cat#D190090;Application:western blot)
γ-H2AX Rabbit polyclonal antibody (Cell Signaling Technology; Cat#9718; Clone#20E3; Application:IF,IB)
Nucleolin Rabbit antibody (Abcam; Cat#ab129200; Clone#EPR7952; Application:IF,IB)
Nucleolin Rabbit antibody (Abcam; Cat#ab22758; Application:RIP)
Fibrillarin Mouse antibody (Abcam; Cat#ab4566; Clone#38F3; Application:IF,IB)
Rad51 mouse antibody (Abnova; Cat#H00005888-B01P;Application:IF,IB)
Histone H3 Rabbit antibody (Wanlei; Cat#WL02243; Application:IB)
PCNA mouse antibody (Cell Signaling Technology; Cat#2586S; Clone#PC10; Application:IF)
RPA32 Rabbit antibody (Abcam; Cat#ab76420; Application:IP, IB)
dsDNA marker mouse antibody (Santa Cruze; Cat#SC-58749; Application:IB)
Cleaved Mouse Caspase-1 (p20) antibody (Adipogen; Cat#AG-20B-0042;Application:IB)
IL-1 beta Rabbit antibody (Abcam; Cat#ab82558; Application:IB)
TMEM173 / STING Rabbit antibody (Lifespan; Cat#LS-B7237; Application:IB)
TBK1/NAK Rabbit antibody (Cell Signaling Technology; Cat#38066; Clone#E8I3G; Application:IB)
Phospho-TBK1/NAK (Ser172) Rabbit antibody (Cell Signaling Technology; Cat#5483S; Clone#D52C2; Application:IB)
IRF-3 Rabbit antibody (Cell Signaling Technology; Cat#4302S; Clone#D83B9; Application:IB)
Phospho-IRF-3 (Ser396) Rabbit antibody (Cell Signaling Technology; Cat#29047S; Clone#D6O1M; Application:IB)
Goat anti-Rat IgG Secondary Antibody, Alexa Fluor Cy3 (Thermo Fisher Scientific; Cat#A10522; Application:IF)
Goat anti-Mouse IgG Secondary Antibody, Alexa Fluor 488 (Thermo Fisher Scientific; Cat#A11029; Application:IF)
Goat anti-Rabbit IgG Secondary Antibody, Alexa Fluor 647 (Thermo Fisher Scientific; Cat#A27040; Application:IF)
Goat anti-Rabbit IgG Secondary Antibody, Alexa Fluor 488 (Thermo Fisher Scientific; Cat#A11034; Application:IF)
Donkey anti-Rabbit IgG Secondary Antibody, Alexa Fluor 555 (Thermo Fisher Scientific; Cat#A31572; Application:IF)
Goat anti-Mouse IgG (H+L) Secondary Antibody, HRP (Thermo Fisher Scientific; Cat#31430; Application:IB)
Goat anti-Rabbit IgG (H+L) Secondary Antibody, HRP (Thermo Fisher Scientific; Cat#31460; Application:IB)

Validation

All Cell Signaling antibodies used in our manuscript were validated by the manufacturer (https://www.cellsignal.com/about-us/our-approach-process/cst-antibody-validation-principles). All Abcam antibodies used were validated by the manufacturer as well (https://www.abcam.com/primary-antibodies/how-we-validate-our-antibodies). Other antibodies, we also provide the citation or links from the commercial website, where the validation is shown. The applications of the antibodies are listed below:
Rabbit IgG, sigma #I5006, Isotype control (https://www.sigmaaldrich.cn/CN/zh/product/sigma/i5006?context=product).
Mouse monoclonal anti-IdU (BD Biosciences #347580) and Rat monoclonal anti-BrdU (Novus #NB500-169) were validated for IF (1:500) (Zhao et al. 2018, Cell Res).
p-ATR (Ser428) Rabbit antibody, Cell Signaling Technology #2853S, reactivity: Human, Mouse, Rat, Monkey, application: IB (1:1000).
ATR Rabbit polyclonal antibody, Cell Signaling Technology #2790S, reactivity: Human, Monkey, application: IB (1:1000). It was also validated that it reactivated in mouse (Zhao et al. 2018, Cell Res).
Chk1 Mouse monoclonal antibody, Cell Signaling Technology #2360S, reactivity: Human, Mouse, Rat, Monkey, application: IB (1:1000).
P-Chk1(S345) Rabbit monoclonal antibody, Cell Signaling Technology #2348S, reactivity: Human, Mouse, Rat, Monkey, application: IB (1:1000) , IF (1:50), FACS (1:200).
Actb mouse antibody, Abclonal #AC004, reactivity: Human, Mouse, Rat, application: IB (1:10000), IHC (1:100), IP (1:50), IF (1:50). (https://abclonal.com.cn/catalog/AC004)
Gapdh Mouse monoclonal antibody, Sangon Biotech D190090, reactivity: Human, Mouse, Rat, application: IB (1:1000), IHC(1:100). (https://www.sangon.com/productDetail?productInfo.code=D190090).
γ-H2AX Rabbit polyclonal antibody, Cell Signaling Technology #9718, reactivity: Human, Mouse, Rat, Monkey, application: IB (1:1000), IHC (1:480), IF (1:1000), FACS (1:200).
Nucleolin Rabbit antibody, Abcam #ab129200, reactivity: Human, Mouse, Rat, application: IB (1:10000), IHC (1:250), IF (1:1000), FACS (1:2000).
Nucleolin Rabbit antibody, Abcam #ab22758, reactivity: Human, Mouse, Rat, application: IB (1:1000), IP (1:100), IF (1:1000), IHC (1:1000). It was validated that it also could be used for RIP experiment (Fig3d-e).
Fibrillarin Mouse antibody, Abcam #ab4566, reactivity: Human, Mouse, Rat, application: IB (1:1000), IF (1:1000), FACS (1:20).
Rad51 mouse antibody, Abnova #H00005888-B01P, reactivity: Human, Mouse, Rat, application: IB (1:500), IF (1:500). (http://www.abnova.com/products/products_detail.asp?catalog_id=H00005888-B01P)
Histone H3 Rabbit antibody, Wanlei #WL02243, reactivity: Human, Mouse, Rat, application: IB (1:1000). There is no web link for the product.
PCNA mouse antibody, Cell Signaling Technology #2586S, reactivity: Human, Mouse, Rat, Monkey, Bovine, Pig, application: IB (1:2000), IF (1:1000), IP (1:100), IHC (1:16000), FACS (1:2400).
RPA32 Rabbit antibody, Abcam #ab76420, reactivity: Human, Mouse, Rat, application: IB (1:10000), IF (1:50), IP (1:100), IHC (1:100).
dsDNA marker mouse antibody, Santa Cruze #SC-58749, application: IB (1:500), IF(1:500). (https://datasheets.scbt.com/sc-58749.pdf)
Cleaved Mouse Caspase-1 (p20) antibody, Adipogen #AG-20B-0042, reactivity: Mouse, application: IB (1:1000), IHC (1:500), IP (1:200).
IL-1 beta Rabbit antibody, Abcam #ab82558, reactivity: Human, Mouse, application: IB (1:1000), IHC (1:1000).
TMEM173 / STING Rabbit antibody, Lifespan #LS-B7237; reactivity: Human, Mouse, Tree shrew, application: IB (1:1000). It was validated for IB (1:1000) (Xu et al. 2020, J Immunol).
TBK1/NAK Rabbit antibody, Cell Signaling Technology #38066, reactivity: Human, Mouse, Rat, application: IB (1:1000), IF (1:800), IP (1:100), FACS (1:1600).
Phospho-TBK1/NAK (Ser172) Rabbit antibody, Cell Signaling Technology #5483S, reactivity: Human, Mouse, application: IB (1:1000), IF (1:50), IP (1:50), FACS (1:50).

IRF-3 Rabbit antibody, Cell Signaling Technology #4302S, reactivity: Human, Mouse, Rat, Monkey, application: IB (1:1000), IP (1:50). Phospho-IRF-3 (Ser396) Rabbit antibody, Cell Signaling Technology #29047S, reactivity: Human, Mouse, Rat, application: IB (1:1000), IP (1:50), IF (1:200), FACS (1:200).

Eukaryotic cell lines

Policy information about cell lines

Cell line source(s)	Wild type mouse ESCs, NSCs and MEF were home made; HR and NHEJ reporter cell lines were from Professor An-yong Xie in Zhejiang University School of Medicine. HEK293T cells used for virus package were the department stocks, originally obtained from ATCC.
Authentication	Not authenticated.
Mycoplasma contamination	All tested negative for mycoplasma.
Commonly misidentified lines (See ICLAC register)	No commonly misidentified cell lines were used in the study.

Animals and other organisms

Policy information about studies involving animals; ARRIVE guidelines recommended for reporting animal research

Laboratory animals	Discn knockout mice were generated using C57BL/6 strain. All behavioral tests were performed on mice from littermates. 8 weeks old female pregnant CD1 mice were used for mouse embryonic fibroblasts isolation. Mice were housed in accordance to the Animal Care and Use Committee rules and guidelines of the Kunming Institute of Zoology, Chinese Academy of Sciences. Mice were kept under 12 h dark/light cycle, with daylight from 8:00-20:00, ambient temperature 20-25 ℃, and 40%-70% humidity.
Wild animals	This study did not use wild animals.
Field-collected samples	This study did not involve samples collected from the field.
Ethics oversight	All works on mice were carried out in accordance with the guidelines of the Animal Care and Use Committee of the Kunming Institute of Zoology, Chinese Academy of Sciences.

Note that full information on the approval of the study protocol must also be provided in the manuscript.

Flow Cytometry

Plots

Confirm that:

☒ The axis labels state the marker and fluorochrome used (e.g. CD4-FITC).

☒ The axis scales are clearly visible. Include numbers along axes only for bottom left plot of group (a 'group' is an analysis of identical markers).

☒ All plots are contour plots with outliers or pseudocolor plots.

☒ A numerical value for number of cells or percentage (with statistics) is provided.

Methodology

Sample preparation	To examine the proliferation, cells were labeled with 10 mM BrdU for 30 min followed by fixation in 4% formaldehyde for 10 min and denaturation in 2 M HCl at room temperature for 1 h and staining with anti-BrdU primary antibody and Cy3 conjugated secondary antibody.For DNA repair assay, mESC reporters were co-transfected with pcDNA3β-I-SceI and GFP or RFP constitutive expression plasmid using Neon transfection system.
Instrument	FACS experiments were performed using a FACS LSRFortessa flow cytometer.
Software	The data were acquired using BD FACSDiva software then analyzed with Flowjo software.
Cell population abundance	Immunophenotyping acquisition was performed on a minimum of 10000 cells.
Gating strategy	BrdU+ cells were gated from FSC-A versus PE-A. HR-RFP cells were defined based on RFP expression within the GFP population. NHEJ-GFP cells were defined based on GFP expression within the RFP population.

☒ Tick this box to confirm that a figure exemplifying the gating strategy is provided in the Supplementary Information.
